# Mechanism of the fungal-like particles in the inhibition of adipogenesis in 3T3-L1 adipocytes

**DOI:** 10.1038/s41598-021-98385-y

**Published:** 2021-09-22

**Authors:** Chanawee Jakkawanpitak, Masashi Inafuku, Hirosuke Oku, Nongporn Hutadilok-Towatana, Ruthaiwan Bunkrongcheap, Natthawan Sermwittayawong, Piyapat Aiemchareon, Decha Sermwittayawong

**Affiliations:** 1 Division of Health and Applied Sciences, Faculty of Science, Prince of Songkla University, Hat Yai, 90110 Songkhla Thailand; 2 Faculty of Agriculture, University of the Ryukyus, 1 Senbaru, Nishihara, Okinawa 903-0213 Japan; 3 Molecular Biotechnology Group, Center of Molecular Biosciences, Tropical Biosphere Research Center, University of the Ryukyus, 1 Senbaru, Nishihara, Okinawa 903-0213 Japan; 4 College of Innovation and Management, Songkhla Rajabhat University, Muang District, Songkhla, 90000 Thailand; 5 Division of Biological Science, Faculty of Science, Prince of Songkla University, Hat Yai, 90110 Songkhla Thailand; 6 Functional Food and Nutrition Program, Faculty of Agro-Industry, Prince of Songkla University, Hat Yai, 90110 Songkhla Thailand

**Keywords:** Obesity, Polysaccharides

## Abstract

The dynamic ability of adipocytes in adipose tissue to store lipid in response to changes in the nutritional input and inflammatory elicitors has a major impact on human health. Previously, we established laminarin-coated beads or LCB as an inflammatory elicitor for adipocytes. However, it was not clear whether LCB inhibits lipid accumulation in adipocytes. Here, we show that LCB acts in the early stage of adipogenesis through both interleukin-1 receptor-associated kinases (IRAK) and spleen tyrosine kinase (SYK) pathways, resulting in the activation of the AMP-activated protein kinase (AMPK) and nuclear factor-κB (NF-κB) complexes, which subsequently cause cell cycle arrest, downregulation of the key transcription factors and enzymes responsible for adipogenesis, inhibition of adipogenesis, and stimulation of an inflammatory response. While LCB could effectively block lipid accumulation during the early stage of adipogenesis, it could stimulate an inflammatory response at any stage of differentiation. Additionally, our results raise a possibility that toll-like receptor 2 (TLR2) and C-type lectin domain family 7 member A (CLEC7A/Dectin-1) might be potential β-glucan receptors on the fat cells. Together, we present the mechanism of LCB, as fungal-like particles, that elicits an inflammatory response and inhibits adipogenesis at the early stage of differentiation.

## Introduction

Adipose tissue is a complex immune organ that contains many cell types, such as adipocytes, fibroblasts, preadipocytes, endothelial cells, and immune cells. Not only does this tissue function to store fat, but it also secretes adipokines which are hormones, cytokines, and small molecules that regulate the whole-body energy homeostasis. Adipose tissue stores fat in adipocytes, which are derived from precursor cells (preadipocytes) that undergo the differentiation process called adipogenesis. Proper adipogenesis has a major implication on human health. Inhibition of adipogenesis may result in metabolic conditions, such as insulin resistance, and type-2 diabetes^1^.

The mechanism of adipogenesis is understood in detail. The in vitro differentiation of preadipocytes (e.g., 3T3-L1 cell line) is initiated by the addition of hormonal inducers, which include insulin, IBMX (1-methyl-3-isobutylxanthine), and dexamethasone^2^. IBMX, a synthetic glucocorticoid, stimulates the glucocorticoid pathways and activates CCAAT/enhancer-binding protein beta (*C/ebpβ*) gene expression, whereas dexamethasone/DEX upregulates the *C/ebpδ* gene expression^3^. The expression of both C/EBPβ and C/EBPδ marks the early stage of adipogenesis. During the 2nd day of differentiation, C/EBPβ activates the expression of peroxisome proliferator-activated receptor gamma (*Pparγ*), which in turn, stimulates the expression of *C/ebpα*^4,5^. The expression of both *Pparγ* and *C/ebpα* signifies the intermediate stage of differentiation. During the intermediate stage, some lipid accumulation in the cells can be observed. The collaboration of both PPARγ and C/EBPα is critical for the expression of enzymes involved in triglyceride synthesis in the late stage or terminal differentiation, such as the fatty-acid synthase (FAS), lipoprotein lipase (LPL), sterol regulatory element-binding protein 1 (SREBP-1), and fatty acid binding protein 4 (FABP4)^3^.

Adipogenesis can be inhibited by several molecules or compounds. One of which, β-glucan, a polymer of β-glucose, was shown to inhibit this process. For example, it was shown that high-molecular-weight barley β-glucan suppressed lipid accumulation in 3T3-L1 adipocytes by downregulating both mRNAs and proteins of the key adipogenesis transcription factors such as PPARγ and C/EBPα^6^. Similarly, bacterial β-glucan from *Aureobasidum* sp. was shown to inhibit adipogenesis in 3T3-L1 adipocytes by decreasing the expression of *Pparγ*, acetyl-CoA carboxylase (*Acc*), and *Fabp4* genes^7^. Additionally, particulate or insoluble β-1,3-glucan could strongly inhibit adipogenesis in 3T3-L1 adipocytes^8^. Taken together, these data suggest that there must be a specific β-glucan receptor on the surface of the cells.

β-glucan receptors and their functions have been investigated mostly from studies done in the context of immune cells. For example, the C-type lectin domain family 7 member A (CLEC7A) or Dectin-1, which contains an immunoreceptor tyrosine-based activation (ITAM)-like motif in its intracellular domain, is a β-glucan receptor for many immune cells, especially in macrophages^9^. CLEC7A/Dectin-1 has been shown to work in concert with the toll-like receptor 2 (TLR2) receptor, a member of the toll-like family receptors, in an inflammatory response by macrophages^10^. Interestingly, TLR2 has been suggested as a receptor candidate for β-glucan in 3T3-L1 adipocytes due to its upregulation in the cells upon the treatment with lipopolysaccharide or LPS^11,12^. In addition to TLR2, a recent study suggested the CD36, a scavenger receptor, to be a β-glucan receptor on both macrophages and 3T3-L1 adipocytes^13^. While the structure and function of β-glucan receptors in the immune cells have been well characterized, it remains unclear what the β-glucan receptor is on the 3T3-L1 adipocytes.

We have recently shown that both differentiating 3T3-L1 adipocytes produce an inflammatory response against zymosan and heat-killed yeast, suggesting the ability of 3T3-L1 adipocytes to respond to the actual fungal particles^14^. A previous study suggested that β-glucan on *Candida albicans* is required for the response of neutrophils^15^, suggesting that β-glucan on fungal particles may activate an inflammatory response in 3T3-L1 adipocytes. However, the fact that the actual fungal particles contain many types of polysaccharides on their cell wall complicates data interpretation. To circumvent this problem, we created fungal-like particles by conjugating laminarin, which is a small-size β-glucan, with polystyrene beads. These particles, namely laminarin-coated beads or LCB, were used in our study. We found that LCB was a stronger inflammatory activator than the actual fungal particles. This was likely due to the fact that β-glucans on LCB were completely exposed to the cells, whereas the accessibility of β-glucans on the actual fungal cell walls was limited. We showed that LCB activated the canonical nuclear factor-κB (NF-κB) pathway, which led to the expression of pro-inflammatory cytokines in 3T3-L1 adipocytes^14^.

It has been shown that proteins or molecules that stimulate an inflammatory response of 3T3-L1 adipocytes inhibited adipogenesis. For instance, tumor-necrosis factor alpha (TNF-α), an inflammatory mediator that binds to TNF-α receptor (TNFR), was shown to inhibit adipogenesis in 3T3-L1 adipocytes through the activation of the β-catenin pathway, which subsequently downregulated the expression of *Pparγ* and *C/ebpα* gene expression^16^. Additionally, another study showed that interleukin-6 (IL-6) reduced adipogenic capability in preadipocytes isolated from both insulin-sensitive (IS) and insulin-resistant (IR) individuals^17^. Moreover, the impairment in the expression of *Pparγ* and *C/ebpα* genes was observed in preadipocytes isolated from IR individuals^17^. In addition to inflammatory cytokines, lipopolysaccharide (LPS) was shown to stimulate an inflammatory response, inhibit adipogenesis, and reduce the expression of *Pparγ* and *C/ebpα* in 3T3-L1 adipocytes^18,19^. Therefore, these data suggest that the down regulation of *Pparγ* and *C/ebpα* may be a common mechanism of inflammatory stimulators to inhibit adipogenesis.

Because LCB triggers an inflammatory response in 3T3-L1 adipocytes, LCB most likely inhibits adipogenesis. However, no previous study has analyzed the effect of fungal particles or fungal-like particles on 3T3-L1 differentiation and whether the particles would only affect the expression of *Pparγ* and *C/ebpα* genes. Therefore, the LCB was used as a tool to understand how 3T3-L1 adipocytes respond to fungal cell walls. We carried out a series of experiments to test whether LCB inhibits adipogenesis and investigate the potential β-glucan receptor on the 3T3-L1 adipocytes and. The mechanism of how adipocytes receive the signal from LCB is analyzed and discussed in this study.

## Results

### LCB inhibits the differentiation/activation (D/A) medium-induced 3T3-L1 adipogenesis in a dose-dependent manner

**Figure 1:**
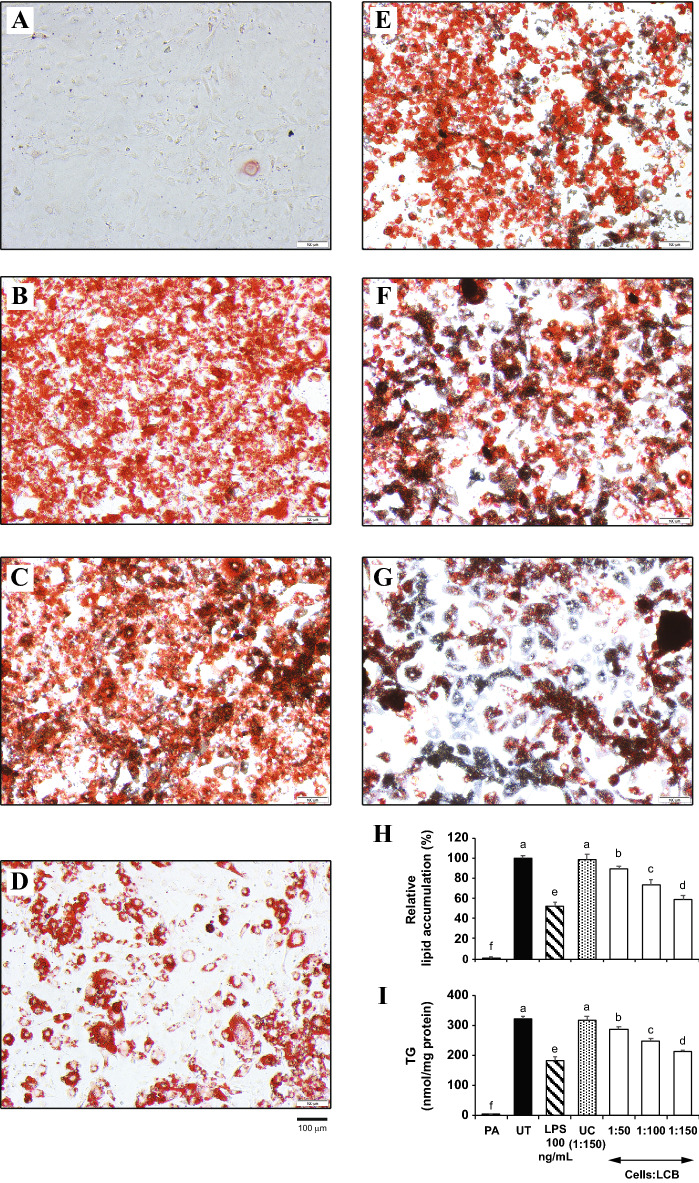
LCB inhibits adipogenesis in a dose-dependent manner. Preadipocytes **(A)**, fully differentiated adipocytes **(B)**, differentiating adipocytes treated with uncoated beads (1:150 cells:UC) **(C)**, 100 ng/mL LPS **(D)**, and an increasing amount of LCB (1:50, 1:100, and 1:150 cells:LCB ratios in **E–G**) were stained with Oil Red O dye, which binds to triglycerides in the cells. LPS, a known adipogenic inhibitor, and LCB were added to the cells from the beginning of differentiation and maintained throughout adipogenesis. **(H)** shows the spectrophotometric absorbance at 520 nm for the Oil Red O dye that binds to triglycerides in the cells. The results are presented as the relative lipid accumulation percentages in respect to the PA set. **(I)** shows the quantitative measurement of the total triglycerides in the cells using the total triglyceride assay. PA, UT, and UC are abbreviated for preadipocytes, untreated, and uncoated beads, respectively.

We hypothesized that LCB may inhibit lipid accumulation in the cells. To test the effect of LCB on adipogenesis, we introduced an increasing amount of LCB to the D/A medium-induced differentiating 3T3-L1 adipocytes. The preadipocytes (Fig. 1A), which do not have lipid accumulation, were included as a negative control, while the fully differentiated adipocytes or the untreated (UT) set (Fig. 1B) served as a positive control for the staining assay. The results show that the percentage of lipid accumulation in the LCB-treated differentiating adipocytes decreases as the amount of LCB added to the cells increases (Fig. 1E–H), suggesting that LCB dose-dependently inhibits adipogenesis. We have previously shown that the cells:LCB ratio of 1:150 did not affect the viability of the fat cells^14^. Thus, a reduction in lipid accumulation in the LCB-treated sets was not due to a decrease in cell viability. The uncoated beads (UC)-treated differentiating adipocytes reveal a comparable level of lipid accumulation with the untreated set (Fig. 1C,H), suggesting that the effect of LCB was conferred by laminarin (β-glucan) on the beads but not the beads themselves. In addition, lipopolysaccharide (LPS) which was previously shown to inhibit the differentiation of 3T3-L1 adipocytes^18^, shows a significant reduction in lipid accumulation (Fig. 1D,H). Therefore, LCB confers a similar adipogenic inhibitory effect to LPS.

To further confirm this effect of the LCB, the total amount of triglyceride (TG) accumulation in adipocytes was determined using a triglyceride (TG) assay. Consistent with the Oil Red O staining results, LCB inhibits the accumulation of triglycerides in 3T3-L1 adipocytes in a dose-dependent manner (Fig. 1I). Collectively, these data suggest that LCB inhibits adipogenesis in a dose-dependent fashion.

### LCB attenuates the D/A medium-induced expression and production of the key adipogenic marker genes in differentiating 3T3-L1 adipocytes in dose- and time-dependent manners

To investigate how LCB inhibits adipogenesis, we analyzed the expression of the key adipogenic transcription factors by means of a qPCR assay. The results show that the expression of *Pparγ*, *C/ebpα,* and *Srebp-1* decreases as the amount of LCB added to the differentiating cells increases (Fig. S1B–D), suggesting a dose-dependent effect of the LCB. In addition, we find that LCB dose-dependently inhibits the expression of genes involved in adipogenesis, including *Fas*, *Lpl,* and *Fabp4* (Fig. S1E–G). LPS, which was included as an inhibitor of adipogenesis, could also significantly suppress the gene expression of those transcription factors and adipogenic enzymes (Fig. S1B–G). Unlike the expression of those genes, the expression of *C/ebpβ* is not affected by LPS or an increasing amount of LCB (Fig. S1A). This observation is consistent with the previous study in which a coffee extract, which was found to inhibit adipogenesis, downregulated the protein expression but not the gene expression of *C/ebpβ*^20^. Because C/EBPβ regulates the expression of *Pparγ* and *C/ebpα*^21^, we think the decrease in the expression of those genes is not directly caused by LCB, but rather the effect on C/EBPβ. Because the transcript of *C/ebpβ* remains unchanged, this suggests that LCB may not affect the expression of *C/ebp*β in the transcription and post-transcriptional levels but it may inhibit the protein expression of C/EBPβ in a post-translational level.

**Figure 2:**
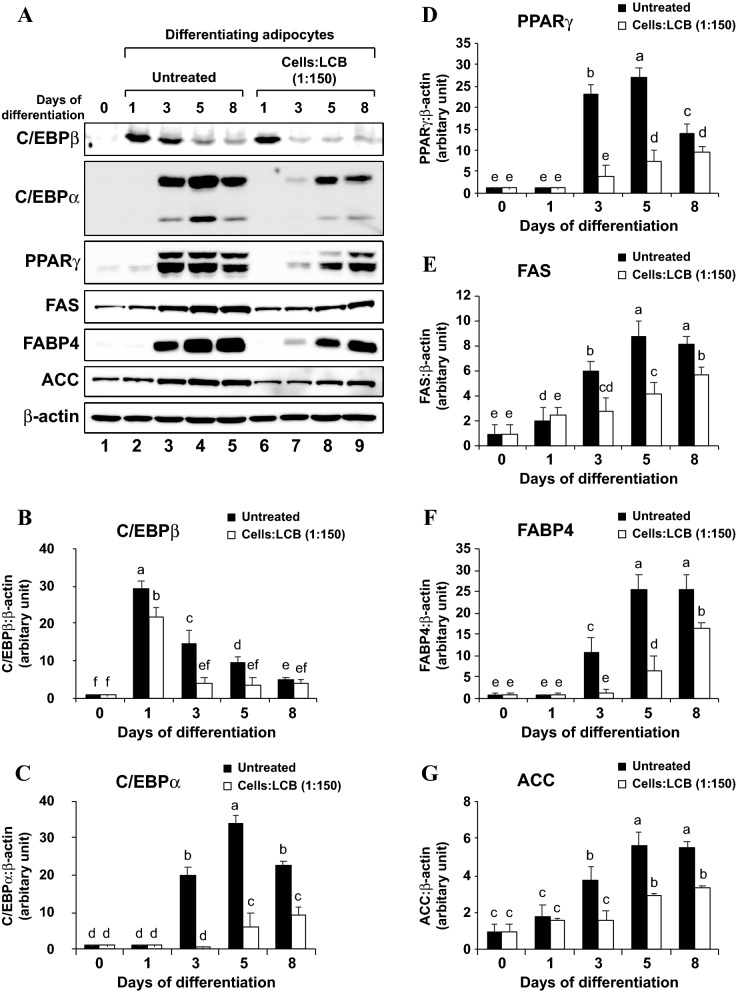
LCB inhibits the expression of key adipogenic transcription factors and enzymes in the differentiating 3T3-L1 adipocytes in a time-dependent manner. **(A)** shows a representative Western blot data set performed using anti-C/EBPβ, anti-C/EBPα, anti-PPARγ, anti-FAS, anti-FABP4, anti-ACC, and anti-β-actin antibodies. The D/A medium, which initiates the differentiation, was added to the cells without or with LCB (1:150 cells:LCB). Cells were collected on days 0, 1, 3, 5, and 8 for the Western blot analysis. β-actin serves as a loading control. **(B–G)** show the expression of C/EBPβ, C/EBPα, PPARγ, FAS, FABP4, and ACC proteins, respectively. The protein expression is calculated from the protein:β-actin ratio from each time point and expressed as the relative ratio in respect to the ratio from day 0 of differentiation shown in lane 1 in **(A)**.

**Figure 3:**
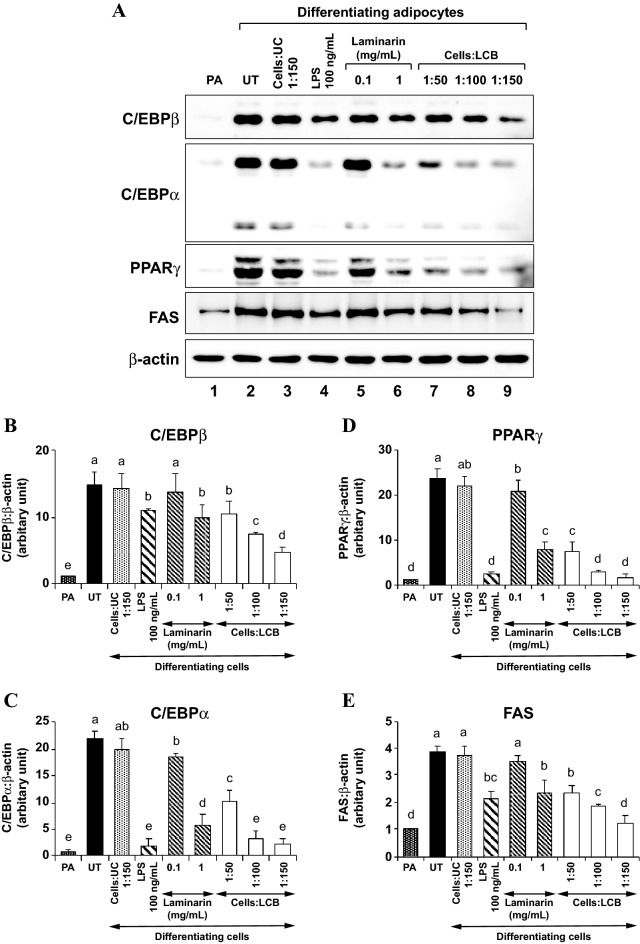
LCB inhibits the expression of key adipogenic transcription factors and enzymes in differentiating 3T3-L1 adipocytes in a dose-dependent manner. **(A)** shows a representative Western blot data set performed using anti-C/EBPβ, anti-C/EBPα, anti-PPARγ, anti-FAS, and anti-β-actin. The D/A medium, which starts adipogenesis, was added to 3T3-L1 preadipocytes without or with uncoated beads (UC) at the ratio of 1:150 cells:UC, 100 ng/mL LPS, soluble laminarin at 0.1 and 1 mg/mL, and an increasing amount of LCB (1:50, 1:100, and 1:150 cells:LCB). UC and LPS serve as a negative and positive control for the inhibition of adipogenesis, respectively. The treatment was performed for 2 days before harvesting the cells for the Western blot assays. **(B–E)** show the expression of C/EBPβ, C/EBPα, PPARγ, and FAS proteins, respectively. The protein expression is calculated from the protein:β-actin ratio from each treatment and expressed as the relative ratio in respect to the ratio from preadipocytes data shown in lane 1 in **(A)**.

To test this hypothesis, we performed Western blotting to analyze the protein level of those adipogenic transcription factors and enzymes in LCB-treated differentiating 3T3-L1 adipocytes. First, we analyzed the kinetic expression of C/EBPβ, C/EBPα, PPARγ, FAS, FABP4, and ACC proteins on days 0, 1, 3, 5, and 8 by means of Western blotting. We find that C/EBPβ protein is expressed as early as day 1 after the start of differentiation and the protein level gradually decreases throughout the course of differentiation, whereas C/EBPα, PPARγ, and FABP4 proteins appear on the 3rd day of differentiation (Fig. 2A,C,F). Although FAS and ACC proteins can be detected even before the differentiation starts, their protein expression is upregulated in the late stage of differentiation (Fig. 2A,E,G). However, the treatment with LCB from the starting period of adipogenesis severely decreased the amount of C/EBPβ, as well as C/EBPα, PPARγ, FAS, FABP4, and ACC proteins (Fig. 2). To further illustrate the effect of LCB on gene expression, we treated the differentiating cells with an increasing dose of LCB for 2 days and analyzed the protein levels of C/EBPβ, C/EBPα, PPARγ, and FAS through Western blotting. We show that an increased amount of LCB results in a reduction of those proteins, suggesting a dose-dependent effect of the LCB (Fig. 3). LPS, which was included as a positive control, also significantly reduces those proteins (Fig. 3). Therefore, these data demonstrate that LCB promotes the reduction in the amount of C/EBPβ protein, causing the downregulation of the other transcription factors and enzymes.

To show that this effect is conferred by β-glucan on the beads, we also treated the cells with uncoated beads and free laminarin. Treatment with uncoated beads does not affect the production of those proteins. In addition, only laminarin at 1 mg/mL concentration reduced protein expression (Fig. 3). As we previously showed that the concentration of laminarin in 1:150 cells:beads in a 48-well was approximately at 4.03 μg/mL^14^, the 1 mg/mL concentration, which causes a noticeable effect to the protein expression, is far more concentrated than the amount of β-glucan on the beads. Therefore, β-glucan must be present on the beads to inhibit adipogenesis through the reduction of C/EBPβ protein, which may subsequently affect gene expression and protein production of other adipogenic genes such as *C/ebpα*, *Pparγ*, *Fabp4*, *Fas*, *Lpl*, and *Srebp-1*.

### LCB suppresses the differentiation of 3T3-L1 adipocytes in the early phase of adipogenesis

**Figure 4:**
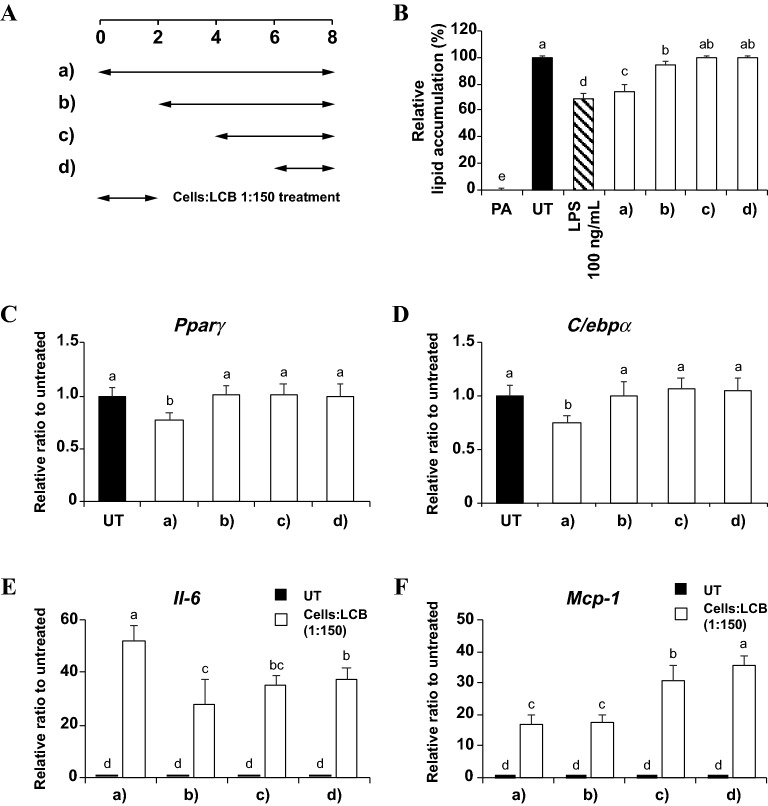
LCB effectively inhibits adipogenesis during the early stage of adipogenic differentiation. **(A)** shows the treatments of differentiating 3T3-L1 adipocytes with LCB at the ratio of 1:150 cells:LCB at different stages of adipogenic differentiation. **(B)** shows the relative lipid accumulation of the treated cells in **(A)** at the end of the differentiation (day 8), using the Oil Red O staining. The set with LPS, performed by treating the cells with LPS throughout the differentiation period (8 days), serves as a positive control for adipogenic differentiation. PA and UT are abbreviated for preadipocytes and untreated, respectively. **(C–F)** show qPCR results for expression of *Pparγ*, *C/ebpα*, *Il-6*, and *Mcp-1* genes, respectively, in differentiating 3T3-L1 adipocytes treated with LCB during the different period of adipogenesis shown in **(A)**. The gene expression of both *Pparγ* and *C/ebpα* genes was analyzed on day 8th, whereas both *Il-6* and *Mcp-1* gene expression was obtained 3 h after the treatment. UT is the untreated set.

The effect of LCB on adipogenesis led us to investigate whether the introduction of LCB at different stages of adipogenesis would equally suppress adipogenesis. Therefore, we treated differentiating adipocytes with LCB (1:150 cells:LCB) at different time periods: day 0, day 2, day 4, or day 6 after the start of differentiation (Fig. 4A). Then, the lipid accumulation and the expression of adipogenic transcription factors (*Pparγ* and *C/ebpα*) of LCB-treated cells at each time period were analyzed by means of the Oil Red O staining and qPCR assays, respectively. We find that LCB could effectively suppress adipogenesis and the expression of the transcription factors when introduced on day 0 but had very little or no effect when the LCB treatment was initiated on day 2 or later (Fig. 4B–D). These results suggest that LCB affects the early stage of adipogenesis, most likely through the reduction of C/EBPβ.

Because LCB can only inhibit adipogenesis in the early stage of differentiation, we wondered if an inflammatory response induced by LCB must also happen in the early stage. To answer this question, we analyzed the expression of *Il-6* and *Mcp-1* genes in differentiating adipocytes treated with LCB by following a similar experimental setup for adipogenesis as described above. After 3 h of treatment with LCB, the results reveal that the expression of *Il-6* and *Mcp-1* genes is relatively high in all conditions and reached the highest levels when LCB was introduced on day 0 and day 6 of differentiation, respectively (Fig. 4E,F). This observation is not unexpected since we showed that LCB induced an inflammatory response in the differentiated 3T3-L1 adipocytes^14^. Therefore, while LCB stimulates an inflammatory response at any stage of adipogenesis, it could only effectively inhibit adipogenesis only at the early stage of differentiation.

### LCB induces cell cycle arrest, suppresses the mitotic clonal expansion (MCE), and downregulates cell cycle-related gene expression during the early phase of 3T3-L1 differentiation

During the early stage of adipogenesis, differentiating adipocytes undergo mitotic clonal expansion (MCE), in which the cells enter approximately 2 rounds of cell division in the first 2 days of differentiation^22^. The cell cycle division is tightly controlled by cyclin and cyclin-dependent kinases (CDK). For example, the G1 to S cycle progression requires the collaboration of cyclin D-CDK4/6 complex^3^. The activation of CDK4/6 leads to phosphorylation of Rb, a negative regulator of E2F proteins, resulting in the release of E2F factors. Free E2F factors promote transcription of cyclin E, an S-phase cyclin. Cyclin E binds CDK2 to promote hyperphosphorylation of Rb to enable the G1/S transition^23^.

**Figure 5:**
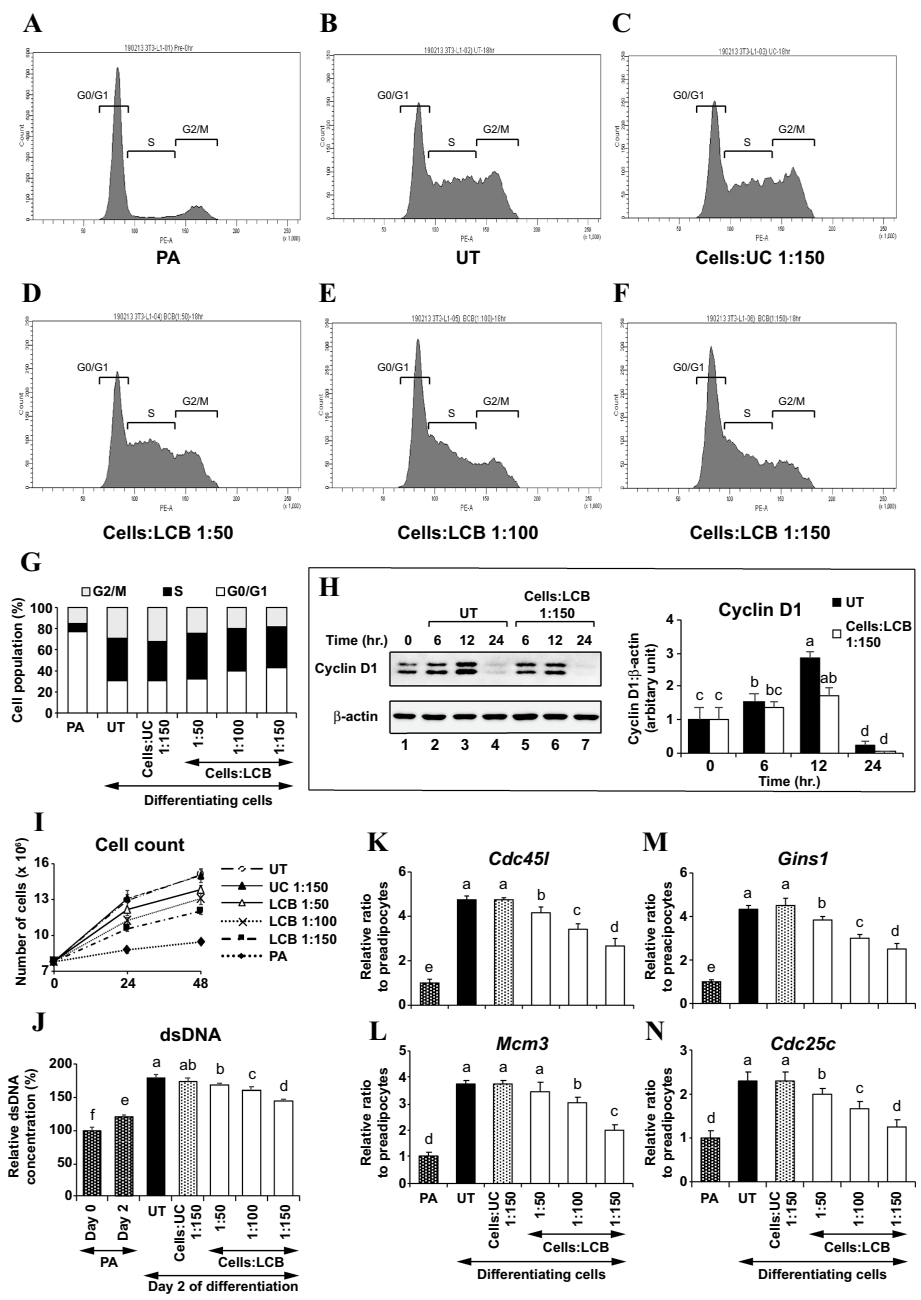
LCB blocks cell cycle progression and inhibits proliferation of differentiating 3T3-L1 adipocytes. **(A–F)** show the flow-cytometry, cell cycle analysis of pre-adipocytes, untreated (UT) adipocytes, uncoated beads (UC)-treated adipocytes, LCB-treated (1:50 cells:LCB) adipocytes, LCB-treated (1:100 cells:LCB) adipocytes, and LCB-treated (1:150 cells:LCB) adipocytes, respectively. Cells were treated without or with LCB from the beginning of adipogenesis induced by the D/A medium as indicated for 18 h, subsequently fixed and stained with Guava cell cycle reagent, and analyzed using a flow cytometer. **(G)** shows the percentage of a cell population in each phase of the cell cycle of the cells analyzed in **(A–F)**. **(H)** shows a representative Western blot data set performed using anti-cyclin D1 and anti-β-actin antibodies. Differentiation of 3T3-L1 adipocytes was induced by adding the D/A medium. The medium was added to the cells without or with LCB at the ratio of 1:150 cells:LCB for 0, 6, 12, and 24 h before being harvested for Western blotting. Western blotting with anti-β-actin antibodies was used as a loading control. The cyclin D1:β-actin protein intensity ratio at each time point was calculated and expressed as the relative ratio in respect to the value from the time 0 (lane 1). **(I)** shows the average cell count in preadipocytes (PA), differentiating 3T3-L1 adipocytes (untreated/UT), differentiating 3T3-L1 adipocytes treated with uncoated beads (UC) at the ratio of 1:150 cells:UC, and differentiating 3T3-L1 adipocytes treated with an increasing amount of LCB at the ratios of 1:50, 1:100, and 1:150 cells:LCB. UC or LCB was added together with the D/A medium at the beginning of differentiation and the cell count was performed at 24 and 48 h after the treatment. **(J)** shows the relative concentration of double-stranded DNA (dsDNA) of pre-adipocytes (PA) grown for 2 days, differentiating 3T3-L1 adipocytes (untreated/UT), differentiating 3T3-L1 adipocytes treated with uncoated beads (UC) at the ratio of 1:150 cells:UC, and differentiating 3T3-L1 adipocytes treated with an increasing amount of LCB at the ratios of 1:50, 1:100, and 1:150 cells:LCB to the dsDNA from PA at day 0. UC or LCB was added together with the D/A medium, which starts adipogenesis, and the relative amount of dsDNA was determined with 48-h treated cells. **(K–N)** show qPCR results for *Cdc45l*, *Mcm3*, *Gins1*, and *Cdc25c* gene expression, respectively. The treatment with UC or LCB was performed as described in **(J)** except that cells were collected for qPCR analysis. Gene expression is reported as the relative gene:β-actin ratio from each treatment in respect to the gene expression from preadipocytes (PA).

Given that LCB inhibits adipogenesis, LCB may affect cell cycle. Previous studies analyzed the effects of adipogenic inhibitors on cell cycle progression and showed that coffee extract, dimethylfumarate, and sulforaphane promoted cell cycle arrest at the G1 phase^20,24,25^. These studies led us to hypothesize that LCB may promote a similar effect to the cell cycle. To test our hypothesis, we investigated the effect of LCB on cell cycle progression in differentiating 3T3-L1 adipocytes by using a flow cytometry technique. Preadipocytes were induced into differentiation with the D/A medium in the absence or the presence of uncoated beads or an increased dose of LCB. While most of the cell population in preadipocytes remains in the G0/G1 phase, the differentiating cells exit the G0/G1 phase and progress into the S and G2/M phases (Fig. 5A,B,G). However, the presence of LCB in the D/A medium dose-dependently increases the number of cells in G0/G1 but decreases the cell population in the G2/M phases (Fig. 5D–G). In addition, we show that LCB inhibits the protein expression of cyclin D1 (Fig. 5H), a factor necessary for G1/S transition^3^. Together, these results suggest that LCB promotes the G0/G1 cell cycle arrest and decreases cyclin D1 protein expression.

The effect on cell cycle progression likely causes a decrease in the total number of differentiated cells. To test this idea, we counted the number of preadipocytes and the D/A medium-stimulated differentiating adipocytes treated without or with the presence of either uncoated beads or an increased dose of LCB for 2 days. We show that the number of preadipocytes does not greatly increase during the 48 h of culturing, confirming that they did not undergo MCE. In contrast, the number of differentiating adipocytes and the differentiating adipocytes treated with the uncoated beads (UC) increase approximately twofold during the 48 h of culturing with the D/A medium, suggesting that treatment with the D/A medium alone stimulates MCE (Fig. 5I). However, the presence of LCB induces a reduction in the total cell number proportionally to the amount of LCB added, suggesting a dose-dependent effect of LCB (Fig. 5I). The loss in the number of the cells is not due to the beads themselves because the uncoated beads produced no effect. To further illustrate the effect of LCB on MCE, we performed an assay to quantify the amount of double-stranded DNA in preadipocytes, differentiating adipocytes, and differentiating adipocytes treated with the uncoated beads or an increased dose of LCB. Consistent with the data in Fig. 5I, an increased dose of LCB causes an increasing loss in the total amount of dsDNA (Fig. 5J).

During the early adipogenesis, the key transcriptional activator C/EBPβ stimulates the expression of many genes involved in DNA replication and cell division, including cell division cycle 45 homolog (*Cdc45l*), mini-chromosome maintenance complex component 3 (*Mcm3*), GINS complex subunit 1 (*Gins1*), and cell division cycle 25 homolog c (*Cdc25c*)^26^. Consistent with this notion, we show that the D/A medium-induced differentiating 3T3-L1 adipocytes (untreated/UT) upregulates the expression of all 4 genes (Fig. 5K–N). Because LCB suppresses the protein expression of *C/ebpβ* (Fig. 2A,B), we think the expression of those cell-cycle regulatory genes must be affected by the treatment with LCB. To test this idea, we analyzed whether LCB would suppress the expression of *Cdc45l*, *Mcm3*, *Gins1*, and *Cdc25c* genes in differentiating adipocytes by means of a qPCR assay. Differentiating 3T3-L1 adipocytes were induced with D/A medium in the presence of the increasing dose of LCB for 48 h prior to being harvested for qPCR analysis. We find that the greater the amount of LCB that is added, the greater the inhibition of the expression of those 4 genes results (Fig. 5K–N), suggesting the dose-dependent inhibitory effect of LCB. Together, these results demonstrate the ability of LCB to inhibit MCE through the promotion of G0/G1 cell cycle arrest and the downregulation of cyclin D1, causing a reduction of the cell number and inhibition of adipogenesis.

### IRAK and SYK signaling pathways mediate an inflammatory-stimulating and adipogenic suppressing effects of LCB on differentiating 3T3-L1 adipocytes

To obtain a clue for which of the β-glucan receptors involved in β-glucan recognition in the differentiating cells, we performed the qPCR assay to check for the expression of *Tlr2*, *Clec7a/Dectin-1*, *Cd36*, and *Tlr4* in differentiating adipocytes treated with LCB. If any of these receptors is important for the cellular response, then their expression should change upon the treatment with LCB. The results show that the expression of *Tlr2* and *Clec7a*/*Dectin-1* genes in the differentiating adipocytes transiently upregulates as the incubation time with LCB progresses from 1 to 24 h (Fig. S2A,B). In addition, we show that the expression of both genes in the cells is increased proportionally to the amount of LCB added (Fig. S2E,F). In contrast, the expression of *Cd36* and *Tlr4* genes remain unchanged upon the treatment with LCB (Fig. S2C,D,G,H). These results suggest that TLR2 and CLEC7A/Dectin-1 but not CD36 and TLR4 receptors are important or may involve in the response of adipocytes to LCB activation.

Previous studies showed that TLR2 and Dectin-1 signaling pathways require interleukin-1 receptor-associated kinase 1 and 4 (IRAK1 and IRAK4) and spleen tyrosine kinase (SYK) for their signal transduction, respectively^27,28^. Therefore, we investigated the involvement of IRAK, SYK, or both proteins in mediating the effect of LCB in differentiating adipocytes by using IRAK 1/4 and SYK inhibitors that inhibit IRAK and SYK proteins, respectively. If IRAK or SYK pathways are required for LCB recognition, then the IRAK1/4, SYK, or both inhibitors should reverse any effect of LCB on the differentiating cells, including an inflammatory response and inhibition of adipogenesis.

We began the analysis by investigating whether the inhibitors could block LCB-induced inflammatory response in the differentiating adipocytes. We find that the expression of nuclear factor kappa B subunit 1 (*Nfkb1*), monocyte chemoattractant protein-1 (*Mcp-1*), prostaglandin-endoperoxide synthase 2 (*Cox-2*), interleukin 6 (*Il-6*), and inducible nitric oxide synthase 2 (*Nos2*) genes in the differentiating adipocytes, which are induced by the presence of LCB, is significantly suppressed in the presence of either IRAK1/4 or SYK inhibitor (Fig. S3A–E). The IKK-2 inhibitor, which was recently shown to inhibit the ability of the inhibitor of IκB kinase β (IKKβ) to degrade IκBα protein for an inflammatory response in adipocytes triggered by LCB^14^, serves as a positive control and could efficiently prevent the effect of LCB (Fig. S3A–E). To show that IRAK and SYK proteins regulate IκBα protein stability, we performed a Western blotting assay to test whether each of the inhibitors can prevent IκBα degradation. The cells were collected for Western blotting after treating with LCB and the inhibitors for 30 min. The results show that IκBα protein stability is increased when either IRAK1/4 or SYK inhibitor was included in the LCB-treated differentiating adipocytes, and it is further enhanced when both IRAK1/4 and SYK inhibitors were combined (Fig. S3F). Thus, these data demonstrate that the IRAK and SYK proteins mediate the LCB-induced inflammatory response through the NF-κB complex in the differentiating adipocytes.

**Figure 6:**
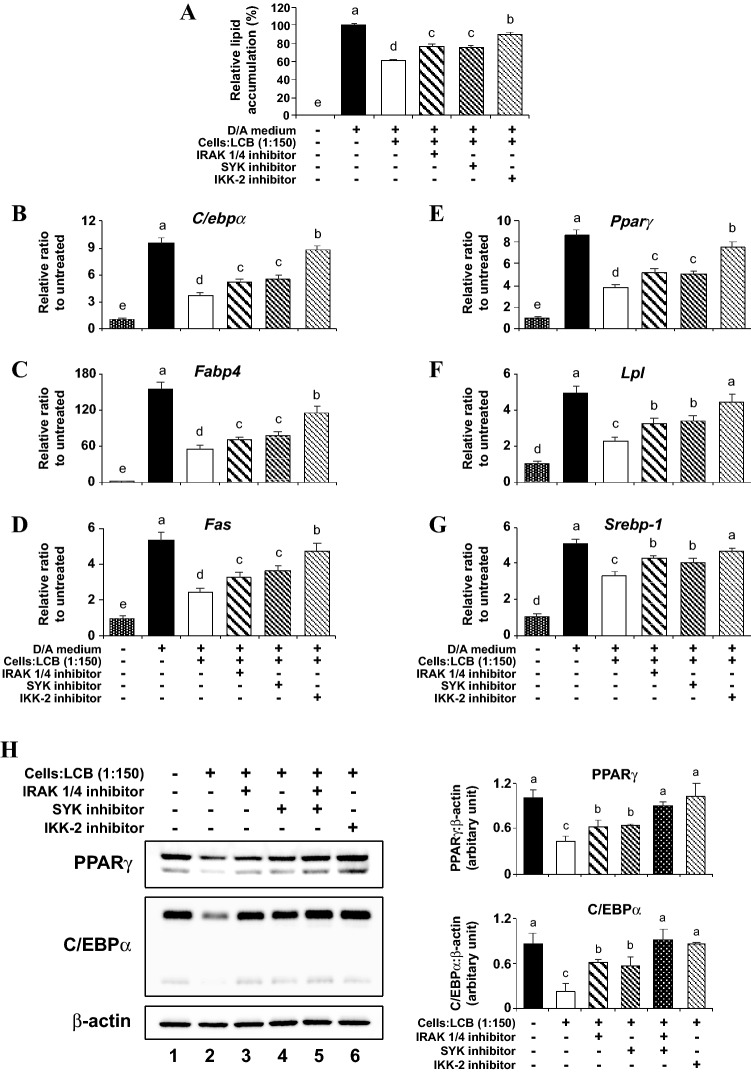
IRAK 1/4 and SYK signaling pathways mediate inflammatory-stimulating and adipogenic suppressing effects of LCB on differentiating 3T3-L1 adipocytes. **(A)** shows the relative lipid accumulation in preadipocytes (without any treatment), D/A-induced differentiating 3T3-L1 adipocytes, D/A-induced differentiating 3T3-L1 adipocytes treated with 1:150 cells:LCB without or with 10 μM IRAK 1/4, 10 μM SYK, both IRAK 1/4 and SKY, or 50 μM IKK-2/SC-514 inhibitor. Preadipocytes were pre-treated with each inhibitor at the indicated concentration for 30 min. Then, the D/A medium together with LCB (1:150 cells:LCB) in the absence or the presence of each inhibitor were added to the cells and maintained throughout the period of adipogenic differentiation (8 days). At the end of differentiation, cells were collected for the Oil Red O staining assay. **(B–G)** show qPCR results for *C/ebpα*, *Fabp4*, *Fas*, *Pparγ*, *Lpl*, and *Srebp-1* gene expression, respectively. Cells were treated as described in **(A)**, except that the treatment with LCB (1:150 cells:LCB) and each inhibitor was performed for 2 days prior to collecting the cells for qPCR analysis. Gene expression is reported as the relative gene:β-actin ratio from each treatment in respect to the gene expression from preadipocytes. **(H)** shows a representative data set for Western blotting assay performed using anti-PPARγ, anti-C/EBPα, and anti-β-actin antibodies with the lysates of cells treated the same way as described in **(B–G)**. The protein expression is calculated from the protein:β-actin ratio from each treatment and expressed as the relative ratio in respect to the ratio from preadipocytes data shown in lane 1 in **(H)**.

To analyze the role of IRAK and SYK signaling pathways in the LCB-triggering inhibition of adipogenesis, we employed the same strategy of using those inhibitors to cripple the pathways. Throughout differentiation (8 days), the presence of either IRAK1/4 or SYK inhibitor significantly increases the lipid accumulation in the LCB-treated differentiating adipocytes, and the amount of lipid accumulation is further increased in the presence of the IKK-2 inhibitor (Fig. 6A). These results suggest that the inhibitors relieve the transcriptional inhibitory effect of LCB on the key markers for adipogenesis. To test this idea, we performed a qPCR assay in the LCB-treated differentiating adipocytes for 2 days, without or with the presence of IRAK1/4, SYK, and IKK-2 inhibitors. We find that the expression of *C/ebpα, Fapb4*, *Fas*, *Pparγ*, *Lpl*, and *Srebp-1*, the key markers for adipogenesis whose expression that are normally repressed by the presence of LCB, are derepressed when either IRAK1/4 or SYK inhibitor is included in the LCB-treated differentiating adipocytes. The derepression is further elevated when the IKK-2 inhibitor was used (Fig. 6B–G). The effects on gene expression most likely led to the effect on protein expression. Therefore, we analyzed the levels of PPARγ and C/EBPα proteins, which are the key proteins for adipogenesis. As expected, the stability of those proteins in the LCB-treated adipocytes is enhanced by either IRAK1/4 or SYK inhibitors. Furthermore, the co-treatment of the IRAK1/4 and SYK inhibitors further enhances the stabilization of PPARγ and C/EBPα (Fig. 6H). Together, these results suggest the role of IRAK and SYK proteins in mediating the inhibitory effect of LCB, including the LCB-mediated inhibition of adipogenesis and LCB-induced inflammatory response through the NF-κB complex.

### LCB activates the AMP-activated protein kinase (AMPK) and acetyl-CoA carboxylase (ACC) phosphorylation at the early stage of adipogenesis

AMPK, a serine/threonine kinase complex, functions in response to a variety of conditions that increase the ratio of AMP/ATP such as exercise, fasting, starvation, and hypoxia^29,30^. This complex consists of a catalytic α-subunit (α1 and α2), a scaffolding β-subunit (β1 and β2) and a regulatory γ-subunit (γ1, γ2, and γ3). Upon the activation by cellular or metabolic stress that increases the AMP/ATP ratio, Thr172 residue on the α-subunit of AMPK is phosphorylated, which stimulates the kinase activity of the complex^31^. Thus, the phosphorylation of AMPK α-subunit at the Thr172 residue is considered the hallmark of AMPK activation. One of the protein targets of AMPK is ACC protein, which catalyzes the formation of fatty acids for adipogenesis^32^. AMPK downregulates the function of ACC enzyme by promoting Ser79 phosphorylation, resulting in the inhibition of adipogenesis^32^.

**Figure 7:**
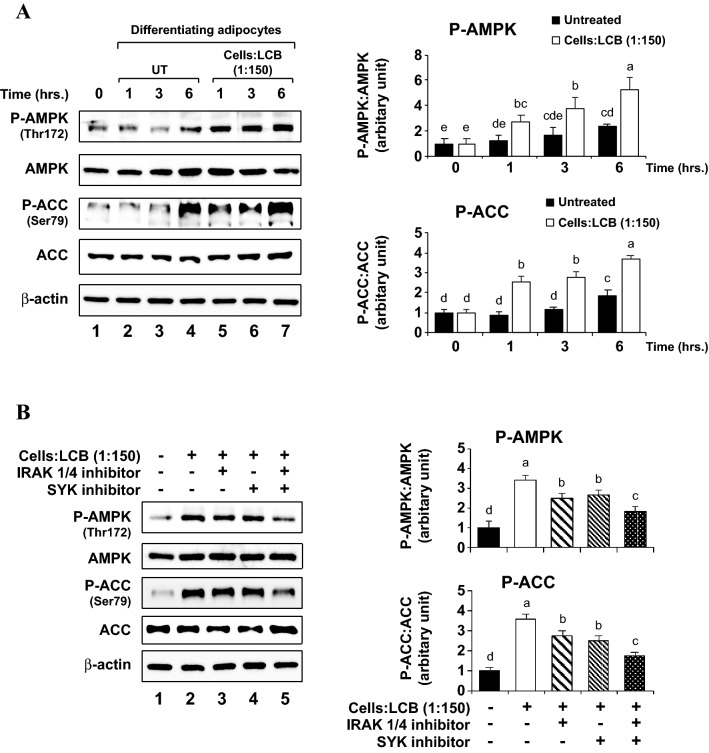
LCB activates AMPK and ACC proteins at the early stage of adipogenesis. **(A)** shows a representative data set for Western blotting assay performed using anti-phospho AMPK (Thr172), anti-AMPK (total), anti-phospho ACC (Ser79), anti-ACC (total), and anti-β-actin antibodies. The D/A medium, which initiates adipogenesis, was added to the cells without or with LCB (1:150 cells:LCB). Treatment was performed for 0, 1, 3, and 6 h prior to harvesting the cells for Western blot analysis. Western blotting with β-actin was performed as a loading control. The protein expression is calculated from the protein:β-actin ratio from each treatment and expressed as the relative ratio in respect to the ratio from preadipocytes data shown in lane 1. **(B)** shows a representative data set for Western blotting assay performed using the same set of the antibodies described in **(A)**. Preadipocytes were pre-treated with either 10 μM IRAK 1/4 or 10 μM SYK inhibitors for 30 min. Then, the D/A medium together with LCB (1:150 cells:LCB) in the absence or the presence of each inhibitor or both were added to the cells, and the incubation was performed for 3 h before harvesting the cells for Western blot analysis. The protein expression analysis was performed as described in **(A)**.

The findings above suggest the role of AMPK as a negative regulator for adipogenesis. To explore whether LCB exerts its adipogenic inhibition through the promotion of AMPK activity, we performed a Western blotting assay to analyze the phosphorylation of AMPK and ACC proteins in the differentiating 3T3-L1 adipocytes with or without the presence of LCB. Consistent with the notion that AMPK inhibits adipogenesis, we find that LCB enhances the Thr172 phosphorylation on the α-subunit of AMPK from the first hour of incubation with LCB and the level of Thr172 phosphorylation is maintained until at least 6 h after the incubation (Fig. 7A). Furthermore, LCB also stimulates Ser79 phosphorylation of the ACC enzymes (Fig. 7A). Thus, these results suggest that LCB may inhibit adipogenesis through the activation of the AMPK complex and phosphorylation of ACC protein at the early stage of adipogenesis.

To analyze whether the activation of AMPK is promoted through the IRAK and SYK kinases, we used the IRAK1/4 and SYK inhibitors with the LCB-treated differentiating adipocytes. We observed a small but significant inhibition of the AMPK and ACC protein phosphorylation; however, the inhibition of the protein phosphorylation is shown when the cells were treated with both IRAK1/4 and SYK inhibitors (Fig. 7B). Thus, these results suggest that both IRAK and SYK signaling pathways mediate the activation of AMPK in the LCB-treated adipocytes.

## Discussion

**Figure 8:**
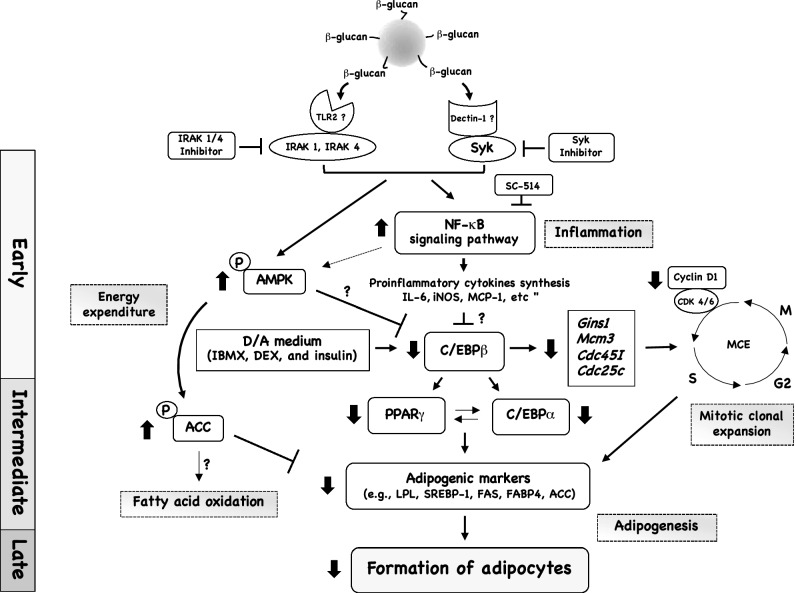
A proposed mechanism of how LCB inhibits lipid accumulation in 3T3-L1 adipocytes. In order for LCB to inhibit adipogenesis in 3T3-L1 adipocytes, LCB must be introduced during the early stage of adipogenic differentiation. In this model, β-glucans on LCB may be recognized by both TLR2 and Dectin-1 receptors on the surface of 3T3-L1 adipocytes. CLEC7A/Dectin-1 transmits its signal through the SYK protein, which can be inhibited by the SYK inhibitor, whereas TLR2 signaling is dependent on IRAK1 or IRAK4 protein, which is blocked by the IRAK 1/4 inhibitor. These two receptors synergistically stimulate the two major pathways: AMPK and the NF-κB signaling pathways. AMPK may inhibit C/EBPβ protein. ACC protein, which is phosphorylated by the AMPK complex, inhibits the expression of key adipogenic markers such as LPL, SREBP-1, FAS, and FABP4. The NF-κB complex, which regulates the expression of proinflammatory cytokines, possibly inhibits the C/EBPβ protein. This protein promotes the expression of other key adipogenic transcription factors genes, including PPARγ and C/EBPα genes. Once the C/EBPβ protein level is attenuated by the presence of LCB, the expression of PPARγ, C/EBPα, and other adipogenic enzymes such as FAS, LPL, and FABP4 are downregulated. The downregulation of adipogenic enzymes and the phosphorylation of ACC protein contributes to a reduction of lipid accumulation in the differentiating adipocytes. In addition, C/EBPβ promotes the expression of cell-division and cell-cycle-related genes such as *Gins1*, *Mcm3*, *Cdc45l*, and *Cdc25c*. The downregulation of C/EBPβ protein affects the MCE, causing a defect in cell division and proliferation. Together, the effect of LCB at the early stage of adipogenesis leads to the downregulation of the key adipogenic enzymes at the intermediate phase of adipocyte differentiation, resulting in the reduction of lipid accumulation in cells.

Recently, we reported that LCB is an inflammatory elicitor for 3T3-L1 adipocytes, which stimulated the expression of many inflammatory-related genes (e.g., *Il-6*, *Mcp-1*, and *Cox-2*) in dose- and time-dependent manners through the activation of the classical NF-κB pathway^14^. In the present study, we further analyzed the mechanistic effect of LCB on differentiating 3T3-L1 adipocytes. We find that upon treatment during the early stage of adipogenesis, LCB promotes cell cycle arrest and inhibits MCE, which results in a significant decrease in cell numbers. Furthermore, LCB suppresses gene and protein expression of the key adipogenic transcription factors and enzymes, plus stimulated phosphorylation of AMPK and ACC proteins, leading to the repression of adipogenesis in 3T3-L1 adipocytes. The mechanistic effect of LCB on the differentiating cells is summarized in Fig. 8.

### Possible β-glucan receptors on 3T3-L1 adipocytes

One of the most important questions that we attempted to answer is what is the specific β-glucan receptor on the cells. Our investigation reveals that the pathways that rely on IRAK and SYK proteins involve the recognition of β-glucan on LCB. We find that any effect that LCB causes to the cells, including an LCB-induced inflammatory response and LCB-mediated inhibition of adipogenesis could be inhibited by the IRAK1/4 or SYK inhibitors. IRAK proteins (IRAK1, IRAK2, IRAK3, and IRAK4) are serine-threonine kinases involved in toll-like receptors (TLRs) and interleukin-1 (IL-1R) signaling pathways^33^. Upon the activation of TLRs or IL-1R, IRAK4 recruits IRAK1 to the receptors, and the kinase activities of these two IRAK proteins are required for the activation of mitogen-activated protein kinases (MAPKs) and the production of TNF-α in macrophages^34,35^. In addition to the IRAK proteins, SYK is a kinase protein required for the receptors that contain the immunoreceptor tyrosine activation (ITAM) domains, such as the classical immune receptors (e.g., Fc-gamma receptor/FcγR) and the c-type lectin receptors (e.g., CLEC7A/Dectin-1 and CLEC2)^36^. SYK protein was previously shown to mediate CLEC7A/Dectin-1 signaling for activation of NF-κB complex and a release of TNF-α in human peripheral blood mononuclear cells^37,38^. Thus, both IRAK and SYK proteins regulate the production of inflammatory cytokines.

Although IRAK and SYK proteins function in multiple receptors, only TLR2 and CLEC7A/Dectin-1, respectively, are the IRAK- and SYK-dependent receptors that bind to β-glucan^39^. In the context of immune cells, it was shown that CLEC7A/Dectin-1 synergized with TLR2 for the degradation of IκBα, and the production of TNF-α^37^, and this synergy requires both CLEC7A/Dectin-1 and TLR2 to be within close proximity^40^. Consistently, either IRAK1/4 or SYK inhibitors suppress the effect of LCB and the enhanced inhibitory effects were observed when both inhibitors were added together (Figs. 6 and 7), illustrating that the IRAK- and SYK-dependent pathways synergize with each other. Thus, the synergy between IRAK and SYK pathways resembles the cooperation between Dectin-1 and TLR2 signaling pathways in the immune cells, suggesting Dectin-1 and TLR2 may be the receptors for β-glucan on differentiating adipocytes.

Interestingly, a recent report suggested cluster of differentiation 36 (CD36), a scavenger receptor, as a candidate for a β-glucan receptor on the surface of macrophages and differentiated adipocytes^13^. CD36 plays a major role as a receptor that mediates the uptake of fatty acids in skeletal muscles and adipose tissues^41^. In addition, CD36 was reported as a β-glucan receptor for zymosan recognition in J744 and human monocyte U937 cells^42,43^. However, we find that the expression of CD36 and TLR4 receptors in the cells was not affected by the incubation with LCB (Fig. S3), suggesting that both CD36 and TLR4 may not involve in the recognition of β-glucans in the cells. Despite the discrepancy, our results did not rule out the possibility that the CD36 might be a β-glucan receptor for the differentiated cells because our study was conducted using the differentiating cells. Therefore, based on the data from our study, at least in the context of the differentiating 3T3-L1 adipocytes, Dectin-1 and TLR2 might play a role in β-glucan recognition. Nonetheless, future experimentation is required to identify the β-glucan receptor on both differentiating and differentiated adipocytes.

### Mechanism of LCB inducing an inflammatory response and adipogenic inhibition in differentiating 3T3-L1 adipocytes

Inflammatory response and adipogenesis are the two related events. We show that LCB can trigger an inflammatory response in differentiating adipocytes at any stage of differentiation (Fig. 4). Other inflammatory elicitors such as TNF-α and LPS can inhibit adipogenesis^18,44^. Unlike an inflammatory response, LCB could only efficiently inhibit adipogenesis when it is introduced at the early stage of differentiation (Fig. 4). Consistently, many compounds or extracts were reported to effectively inhibit adipogenesis when they were incubated with the differentiating cells from the beginning^45–48^. These results suggest that many adipogenic inhibitors may share a common target, which is probably active during the early stage of adipogenesis. Additionally, we show that the IKK-2/SC-514, which inhibits IKKβ, efficiently reverses the effects of LCB (Fig. 6 and Fig. S3), suggesting that the NF-κB complex regulates both inflammation and adipogenesis in adipocytes.

The most likely explanation for the mechanism of how LCB inhibits adipogenesis and stimulates inflammation is that it activates the NF-κB complex, which subsequently targets the C/EBPβ, a key transcription factor for adipogenesis. At the early stage of adipogenesis, C/EBPβ is expressed rapidly after the hormonal inducers from day 1, whereas PPAR and C/EBPα appear from day 3 onward (Fig. 2). This observation is consistent with the previous finding that C/EBPβ is required for the subsequent induction of PPARγ and C/EBPα^49^. By treating the differentiating adipocytes with LCB at the early stage of adipogenesis, the C/EBPβ protein but not mRNA expression is significantly inhibited (Figs. 2 and 3), leading to a downregulation of the major adipogenic transcription factors and enzymes (e.g., PPARγ, C/EBPα, FAS, and FABP4) (Figs. 2, 3, and Fig. S1). It was shown that C/EBPβ forms a dimer to stabilize itself through the basic leucine-rich zipper domain (b-zip)^50^. Thus, LCB may cause instability to the C/EBPβ dimer or promote the monomeric form of C/EBPβ, causing C/EBPβ to be degraded via the ubiquitin–proteasome pathway^50^. Nonetheless, exactly how LCB promotes the protein degradation of C/EBPβ requires further investigation.

C/EBPβ also plays an important role in the mitotic clonal expansion (MCE)^51^. A previous study showed that mouse embryo fibroblasts (MEFs) from C/EBPβ-deficient mice could not undergo MCE, as well as adipogenesis^51^. Similarly, the RNA interference (RNAi)-mediated knockdown of C/EBPβ also inhibited MCE and differentiation of 3T3-L1 adipocytes^52^, suggesting the requirement of C/EBPβ for MCE and adipogenesis. These data are consistent with our finding that LCB inhibits MCE in differentiating 3T3-L1 cells by blocking the cell cycle at the G1/S transition through suppressing the expression of cell cycle regulatory proteins (e.g., cyclin D1) (Fig. 5). Thus, it is highly likely that LCB exerts the inhibitory effects on MCE and adipogenesis at the early stage of differentiation, mainly by promoting the degradation of C/EBPβ protein.

In addition to C/EBPβ, LCB acts through the AMPK protein complex by promoting the T172 phosphorylation at the α-subunit (Fig. 7A). AMPK activation using 5-aminoimidazole-4-carboxamide ribonucleoside (AICAR) inhibited adipogenesis^53^. Furthermore, AMPK activation by metformin or A769662 in mouse embryonic fibroblast (MEF) resulted in the inhibition of adipogenesis and the suppression of C/EBPβ protein^54^, suggesting a mechanism of how AMPK inhibits adipogenesis. Consistent with this notion, a previous work done in hepatoma cells showed that C/EBPβ was suppressed by AICAR^55^, further strengthening a connection between C/EBPβ and AMPK. Based on our data, AMPK is activated within the first hour upon LCB exposure (Fig. 7A), whereas C/EBPβ protein level is upregulated within the first day of differentiation (Fig. 2). Thus, when LCB was added at the early stage of adipogenesis, it stimulated AMPK, which subsequently inhibits C/EBPβ protein. LCB also induces the production of inflammatory cytokines through the activation of NF- κB complex. These inflammatory cytokines and the NF-κB complex may contribute to the inhibition of C/EBPβ in the cells. A future experiment is required to elucidate the relationship among NF-κB, AMPK, and C/EBPβ proteins in the LCB-treated differentiating adipocytes.

The notion that AMPK inhibits adipogenesis remains a controversy. A previous work by Wang and colleagues analyzed the effect of LPS on adipogenesis in 3T3-L1 adipocytes and showed that LPS inhibited AMPK Thr172 phosphorylation, suggesting a positive role of AMPK in adipogenesis^18^. Despite the differences, we noticed that Wang and colleagues investigated AMPK phosphorylation in the time period throughout the course of differentiation (day 0–8) and began to detect the lower level of AMPK Thr172 phosphorylation from day 2 onward, suggesting the role of AMPK in the intermediate and late stages of adipogenesis. In contrast, we analyzed the phosphorylation of AMPK during the first 6 h of adipogenesis (Fig. 7), demonstrating the role of AMPK in the early stage of differentiation. Therefore, these data together suggest that AMPK has a differential effect on adipogenesis, depending on the timing throughout differentiation.

In conclusion, we have illustrated the mechanism of LCB, which was previously shown as an inflammatory elicitor for adipocytes, in the inhibition of adipogenesis and cell cycle progression in differentiating adipocytes. LCB may be recognized by both TLR2 and CLEC7A/Dectin-1 on the cell surface. However, additional experiments will be required to determine the actual β-glucan receptor on the surface of adipocytes.

## Materials and methods

### Preparation of laminarin-coated beads (LCB)

The preparation of LCB was performed using 1,1’-carbonyldiimidazole (CDI)-mediated conjugation as previously described^14^.

### Cell culture and differentiation of 3T3-L1 adipocytes

Mouse 3T3-L1 fibroblasts (ATCC) were cultured in the growth media or Dulbecco’s modified Eagle’s medium–high glucose (DMEM, Sigma-Aldrich) supplemented with 1% Pen-Strep (100 U/mL penicillin and 100 µg/mL streptomycin) and 10% fetal bovine serum (FBS) at 37 °C and 5% CO_2_. 3T3-L1 adipocyte differentiation was performed using the differentiation/activation medium (D/A) as described earlier^14^. In brief, the differentiation was started by incubation of two-day post-confluent cells (defined as Day 0) with the D/A medium containing the hormonal inducers (10 µg/mL insulin, 0.5 mM IBMX (1-methyl-3-isobutylxanthine), and 1.0 µM dexamethasone). On Day 2, the D/A medium was changed to the maintenance medium or D/M (DMEM-high glucose, 10% FBS, 1% Pen-Strep, and 10 µg/mL insulin) and incubated for another 2 days (from Day 3–4). Subsequently, the medium was switched to the growth medium and refreshed every two days until it was fully differentiated to adipocyte (Day 8). The level of differentiation was quantified using the Oil Red O staining and the triglyceride assays.

### Oil Red O staining

To determine lipid accumulation, cells were washed with 1xPBS prior to fixing with 10% formalin solution for 1 h at room temperature. The fixed cells were washed with 60% isopropanol and let dry completely. 0.3% (w/v) Oil Red O solution was added and incubated for 10 min at room temperature. After incubation, cells were washed three times with distilled water to remove the excess dye. Images of the cells were collected using an inverted microscope (CKX53, Olympus). Then, isopropanol was added to dissolve the dye and the absorbance was measured at 520 nm.

### Triglyceride assay

The assay was modified from the previous study^56^. Briefly, after washing with 1xPBS, the cells were extracted using lysis solution (0.05% Triton X-100 in 1xPBS). The whole-cell lysates were then heated up at 70 °C for 10 min and gently vortexed for 30 s or until the sample was completely homogenized. The protein content was measured according to the Bradford assay as previously described^57^. For triglyceride (TG) detection, the assay was set up by mixing the homogenized sample with 250 unit/mL lipase (Sigma-Aldrich) in the ratio of 1:1. After incubation at 37 °C for 60 min, the mixture was centrifuged at 12,000 rpm for 5 min, and the glycerol content in the sample was determined with a free glycerol reagent kit (Sigma-Aldrich) as recommended by the company. The concentration of TG in the sample was calculated by referring to the glycerol standard curve and the TG values were expressed as nmol of TG/mg of protein.

### 3T3-L1 differentiating adipocytes stimulation with LCB

To analyze the effect of LCB on adipocyte differentiation, LCB (1:150 cells:LCB) was added to the cells at different time intervals during the differentiation process as shown in Fig. 4A. The relative percentage of lipid accumulation was analyzed by Oil Red O staining on Day 8 of differentiation. To evaluate the molecular mechanisms of LCB on adipogenesis, a desired concentration of LCB (e.g. 1:150 cells:LCB) was added together with D/A medium to the 3T3-L1 fibroblast cells. Adipocyte differentiation was performed as described above. For gene expression analysis, cells were harvested at 3 and 48 h after treatment for investigating inflammatory genes expression (e.g., *Il-6*) and adipogenic/cell cycle regulatory genes expression (e.g., *Pparγ* and *Cdc45l*), respectively. For determination of adipogenic protein expression, the incubation time (1–8 days) was performed before subjecting the cells to Western blotting assay. The expression of cyclin D1 was observed from 0 to 24 h after the stimulation. The phosphorylation of AMPK and ACC was detected during the first 6 h of adipogenesis, and the degradation of IκBα protein was examined at 30 min after the treatment with LCB.

### Assay with inhibitors

After pre-treating the cells with the selective inhibitors; either SC-514 (50 µM, Sigma-Aldrich), IRAK 1/4 inhibitor (10 µM, Sigma-Aldrich), SYK inhibitor (10 µM, Abcam), or IRAK 1/4 plus SYK inhibitors (10 µM) in the growth medium for 30 min, the medium was removed. Then, a fresh D/A medium containing the same concentration of those inhibitors and LCB (1:150 cells:LCB) was added. Cells were harvested at the indicated time points for analyzing the molecular mechanisms of LCB on adipogenesis, as described above.

### Quantitative PCR (qPCR)

Total RNA extraction and complementary DNA (cDNA) synthesis were performed as previously described^14^. Real-time PCR was conducted using a real-time PCR machine (Stratagene Mx3005P, Agilent Technologies) with EvaGreen fluorescence dye (Solis BioDyne). The primer sequences used for this study are listed in Table S1. All qPCR assays were performed with the annealing temperature at 60 °C for 40 cycles. Data were analyzed using the 2^−ΔΔCt^ method^58^ and β-actin were used as an internal control.

### Western blot analysis

Protein sample preparation was performed similarly as previously described^14^, except that the lysates were prepared with PRO-PREP protein extraction solution (iNtRON Biotechnology). Samples were then resolved on 7.5%, 10%, or 15% SDS–polyacrylamide gel and transferred to a PVDF membrane (0.45 µm, Merck). Primary antibodies against IκBα (Abcam); total AMPKα (Santa Cruz Biotechnology), phospho-AMPKα (Thr 172) (Santa Cruz Biotechnology), phospho-Cdk (Thr14/Tyr15) (Santa Cruz Biotechnology); β-actin (Cell Signaling), C/EBPβ (Cell Signaling), C/EBPα, PPARγ (Cell Signaling), FABP4 (Cell Signaling), FAS (Cell Signaling), ACC (Cell Signaling), phospho-ACC (Ser79) (Cell Signaling), and cyclin D1 (Cell Signaling) were applied with 1:1,000-dilution, except for IκBα and β-actin which were used at the concentration of 1:10,000 and incubated for 12–16 h at 4 °C. After incubating with 1:10,000-diluted horseradish peroxidase (HRP) conjugated secondary antibody (Cell Signaling) for 1 h at room temperature, and the enhanced chemiluminescence analysis system (Wako Chemicals) was used to develop the signal. Images were captured using an ImageQuant LAS 4000 (GE Healthcare). All the signal intensities were quantified by ImageJ 1.49t software.

### Measurement of double-stranded DNA (dsDNA)

To quantify the dsDNA concentration, 3T3-L1 cells were treated with the D/A medium in the presence or absence of LCB (1:150 cells:LCB) for 48 h. The treated cells were washed twice with 1xPBS prior to lysis with SDS lysis buffer (10 mM Tris–HCl (pH 8), 1 mM EDTA, and 0.05% SDS). After completing homogenization, the mixture was centrifuged at 13,000 rpm 4 °C for 5 min. Only the supernatant was subjected to the dsDNA assay, which was performed using Qubit dsDNA BR Assay Kit (Thermo Fisher Scientific) as recommended by the manufacturer.

### Cell cycle analysis (flow cytometry)

After being treated for 18 h without or with LCB (1:150 cells:LCB) in the D/A medium, 3T3-L1 differentiating cells were detached using the Accutase solution (Innovative Cell Technologies) and fixed with 70% (v/v) cold ethanol at − 20 °C for 24 h. Then, the fixed cells were collected by centrifugation at 1,000 rpm for 3 min. The cell pellet was resuspended with the guava cell cycle reagent (Merck) and incubated for 30 min at room temperature. Subsequently, the cell suspension samples were transferred to a new tube for cell cycle analysis using BD FACSCanto II Flow Cytometry System (BD Bioscience), and the data were analyzed using BD FACSDiva software (BD Bioscience).

### Statistical analysis

All data were performed at least three times independently and expressed as the mean ± S.D. The statistical significance was determined by the statistical software R (version 3.6.2) using one-way ANOVA with Duncan’s multiple range test. Two datasets with different letters are significantly different with *p* < 0.05.

## Supplementary Information

Supplementary Information.

## Data Availability

All data generated or analyzed during this study are included in this published article (and its Supplementary Information files).
