# Supplementary material for: Mechanism of the fungal-like particles in the inhibition of adipogenesis in 3T3-L1 adipocytes
Source: Sci Rep. 2021 Sep 22;11:18869. doi: 10.1038/s41598-021-98385-y (PMC8458348; doi:10.1038/s41598-021-98385-y)
Supplement: Supplementary file 1 — Supplementary Information. [file 41598_2021_98385_MOESM1_ESM.docx]

**Table S1: List of primers**

| **Gene** | **Description** |  | **Sequence (5′ to 3′)** |
| --- | --- | --- | --- |
| ACTB | Actin Beta | **F** | CCTGAGGCTCTTTTCCAGCC |
|  |  | **R** | GCCAGAGCAGTAATCTCCTTCTG |
| iNOS | Inducible nitric oxide synthase | **F** | TTGGAGCGAGTTGTGGATTGTC |
|  |  | **R** | GCAGCCTCTTGTCTTTGACCCAG |
| IL6 | Interleukin 6 | **F** | GAGGATACCACTCCCAACAGACC |
|  |  | **R** | CTGCAAGTGCATCATCGTTGTTC |
| MCP-1 | Monocyte Chemoattractant Protein-1 | **F** | CTCATTCACCAGCAAGATGATCC |
|  |  | **R** | CCTTCTTGGGGTCAGCACAG |
| COX-2 | Cyclooxygenase-2 | **F** | CCTCTGCGATGCTCTTCC |
|  |  | **R** | TCACACTTATACTGGTCAAATCC |
| NF-κB | Nuclear factor kappa-light-chain-enhancer of activated B cells | **F** | TGGCAGACGATGATCCCTACG |
|  |  | **R** | CGGAATCGAAATCCCCTCTGTT |
| TLR2 | Toll-like receptor 2 | **F** | CCGAAACCTCAGACAAAGC |
|  |  | **R** | GAGGACTGTTATGGCCACC |
| TLR4 | Toll-like receptor 4 | **F** | CTCTAGCCCACTGCTTCAGGC |
|  |  | **R** | CCCTGACTGGCACTAACCACAT |
| Clec7a/Dectin-1 | C-type lectin domain family 7 member A | **F** | GACCCAAGCTACTTCCTCA |
|  |  | **R** | GCAGCACCTTTGTCATACTG |
| C/EBPβ | CCAAT/enhancer binding protein (C/EBP) beta | **F** | ACGACTTCCTCTCCGACCTCT |
|  |  | **R** | CGAGGCTCACGTAACCGTAGT |
| C/EBPα | CCAAT/enhancer binding protein (C/EBP) alpha | **F** | TGCGCAAGAGCCGAGATAA |
|  |  | **R** | CGGTCATTGTCACTGGTCAACT |
| PPARγ | Peroxisome proliferator-activated receptor gamma | **F** | CTATGGAGTTCATGCTTGTGAAGG |
|  |  | **R** | CTTCAATCGGATGGTTCTTCGGA |
| FABP4 | Fatty Acid-Binding Protein 4 | **F** | TGGAAGCTTGTCTCCAGTGA |
|  |  | **R** | AATCCCCATTTACGCTGATG |
| FAS | Fatty acid synthase | **F** | TTGGAGCCTGTGTAGCCTTCGAG |
|  |  | **R** | GGTGTGTGAGCCGTCAAACAGG |
| LPL | Lipoprotein lipase | **F** | CCATGGATGGACGGTAACGGG |
|  |  | **R** | CCCGATACAACCAGTCTACTACA |
| SREBP-1 | Sterol regulatory element-binding protein 1 | **F** | AGCCACACTTCATCAAGGCA |
|  |  | **R** | TGGTCCCTCCACTCACCAG |
| Cdc45l | Cell division cycle 45 | **F** | AAGGGGAATCTGCGAGAAATG |
|  |  | **R** | GCCAGGAATTTATGCTTGAAC |
| Mcm3 | Minichromosome maintenance complex component 3 | **F** | CGACGCTACTCTGACCTCAC |
|  |  | **R** | CTGTCTCCAGGGGGTTGTTC |
| Gins1 | GINS complex subunit 1 | **F** | CTGGACGAGGGGATCTGATAC |
|  |  | **R** | CCCATATTCCCACCTGAGTGC |
| Cdc25 | Cell division cycle 25 | **F** | CCATTCAGATGGAGGAGGAAG |
|  |  | **R** | GTTTAAGGCTCCCAGGATGTG |
| CD36 | Cluster of differentiation 36 | **F** | TGGCCTTACTTGGGATTGG |
|  |  | **R** | CCAGTGTATATGTAGGCTCATCCA |

**Fig. S1: LCB inhibits the D/A medium-induced expression of the key adipogenic markers in differentiating 3T3-L1 in a dose-dependent manner. (A), (B), (C), (D), (E), (F),** and **(G)** show a representative qPCR results for *C/ebpβ*, *Pparγ*, *C/ebpα*, *Srebp1*, *Fas*, *Lpl*, and *Fabp4* gene expression, respectively. Differentiation of 3T3-L1 adipocytes was induced by adding the D/A medium. The medium was added to the cells without or with LCB at the increasing amount (1:50, 1:100, and 1:150 cells:LCB ratios). PA (pre-adipocytes) is a negative control set, whereas LPS at 100 ng/mL was included as a positive control for inhibition of adipogenesis. The treatment was performed for 2 days before harvesting the cells for qPCR assay. Each bar is the average value from 3 independent experiments and the error bars represent standard deviations.

**Fig. S2: LCB affects the expression of TLR2 and Clec7a/Dectin-1 but not CD36 and TLR4 genes in differentiating 3T3-L1 adipocytes in time- and dose-dependent manners. (A), (B), (C),** and **(D)** show qPCR results for *Tlr2*, *Clec7a/Dectin-1*, *Cd36*, and *Tlr4* gene expression, respectively. The D/A medium was added to the pre-adipocytes without or with LCB (1:150 cells:LCB). Cells were collected for qPCR assay after 1, 3, 6, and 24 hours of treatment. **(E-H)** also show qPCR results for the same set of genes as in **(A-D)**, except that in the differentiating adipocytes were treated with an increased concentration of LCB (1:50, 1:100, and 1:150 cells:LCB) for 3 hours before being collected for qPCR analysis. LPS at 100 ng/mL was included as a positive control. UT, abbreviated for untreated, is the differentiating cells and serves as a reference for the gene expression.

**Fig. S3: IRAK 1/IRAK 4 and SYK signaling pathways mediate an inflammatory-stimulating effect of LCB on differentiating 3T3-L1 adipocytes.** **(A)**, **(B)**, **(C)**, **(D)**, and **(E)** show qPCR results for *Nfkb1*, *Mcp-1*, *Cox-2*, *Il-6*, and *Nos2*, respectively. Pre-adipocytes were pre-treated with 10 μM IRAK 1/4, 10 μM SYK, or 50 μM IKK-2 inhibitor for 30 minutes. Then, the D/A medium together with LCB (1:150 cells:LCB) in the absence or the presence of each inhibitor were added to the cells, and the incubation was performed for 3 hours before collecting the cells for qPCR assay. **(F)** shows the IκBα protein expression in the D/A medium-induced differentiating 3T3-L1 adipocytes treated with either IRAK 1/4 or SYK inhibitor. The experiment was performed similarly to **A-E**, except that cells were treated with 1:150 cells:LCB for 30 minutes prior to being harvested for the Western blotting assay. The IκBα:β-actin protein intensity ratio at each time point was calculated and expressed as the relative ratio in respect to the value from the differentiating cells without LCB and the inhibitor treatment (lane 1).

**Original Western blot pictures**

**Explanation:** After transferring proteins to a PVDF membrane, the membrane was cut so that the Western blotting experiment with different antibodies could be performed in parallel. Areas presented in the manuscript figures are boxed. Each antibody used in this study is specific. Details for each lane are the same as described in its figure legend.


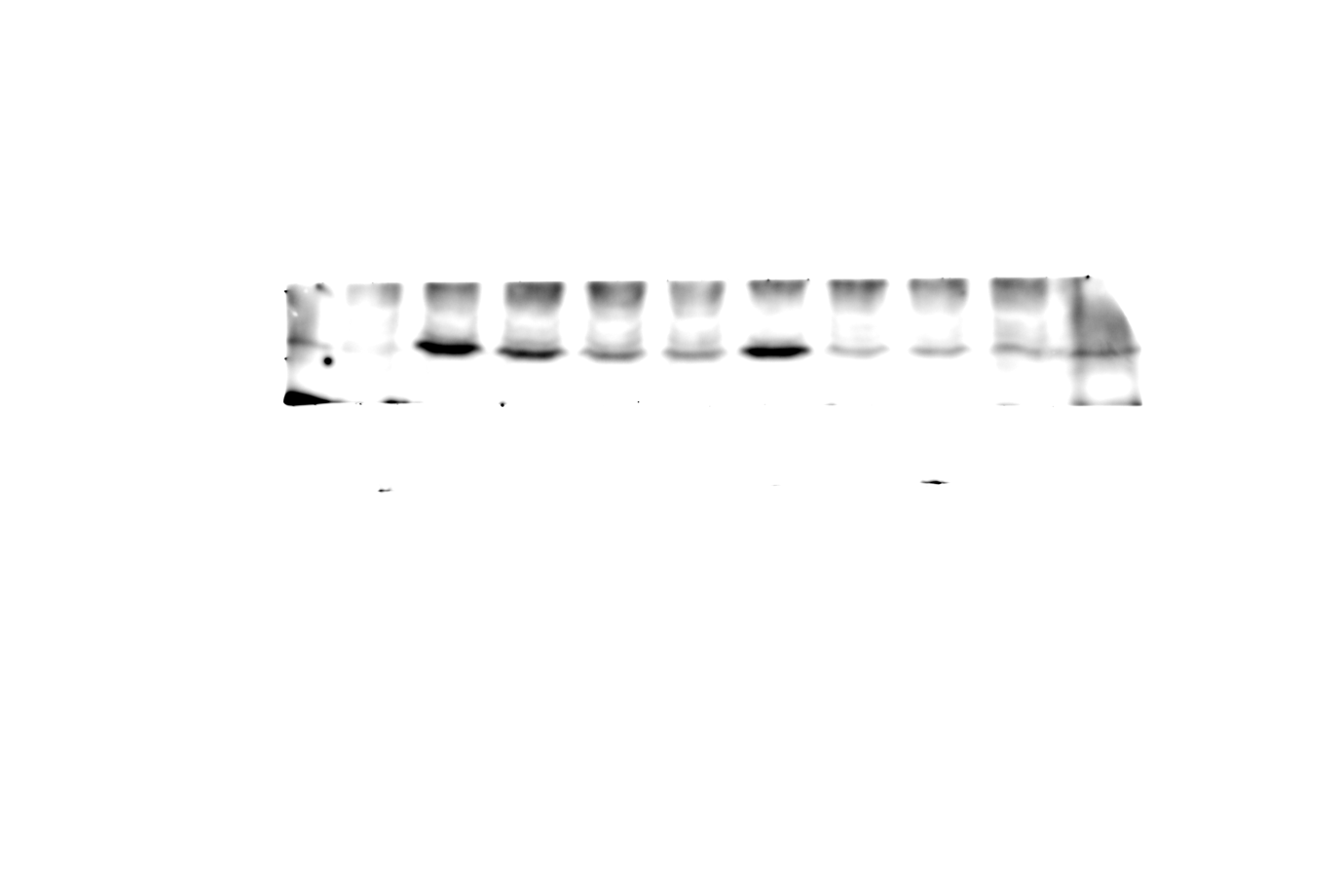


**Fig.2A**

**C\EBPβ**


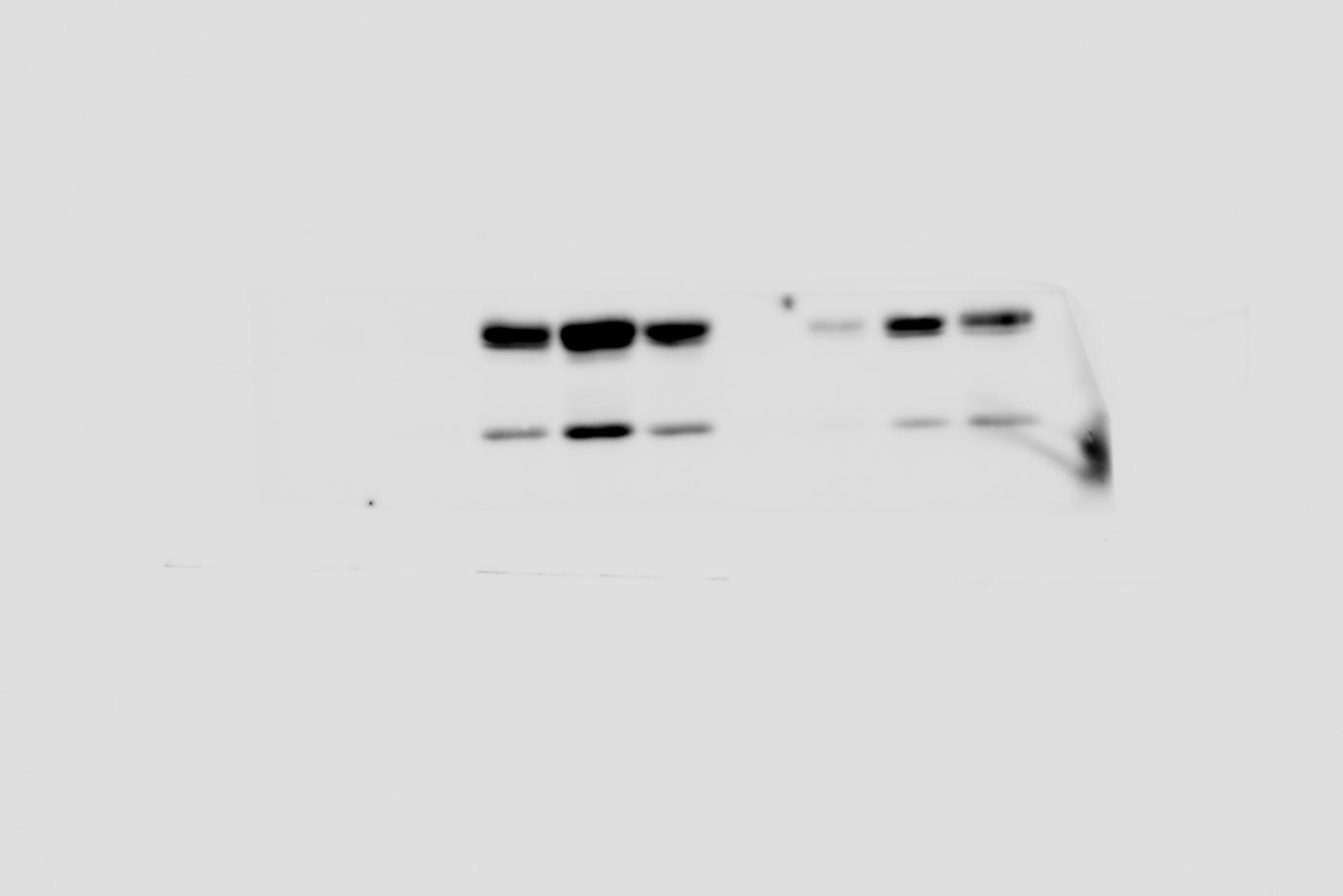


**C\EBPα**


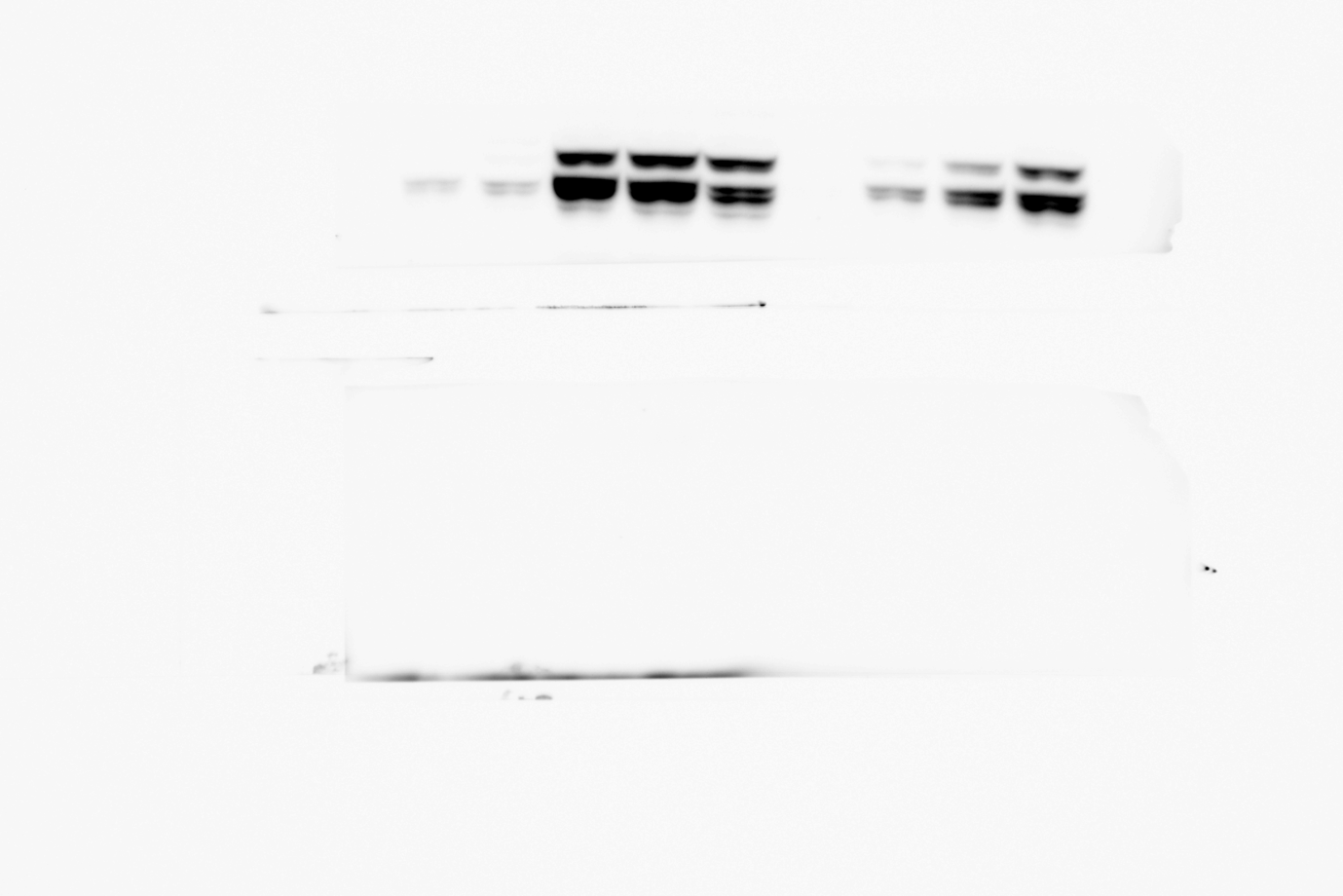


**PPARγ**


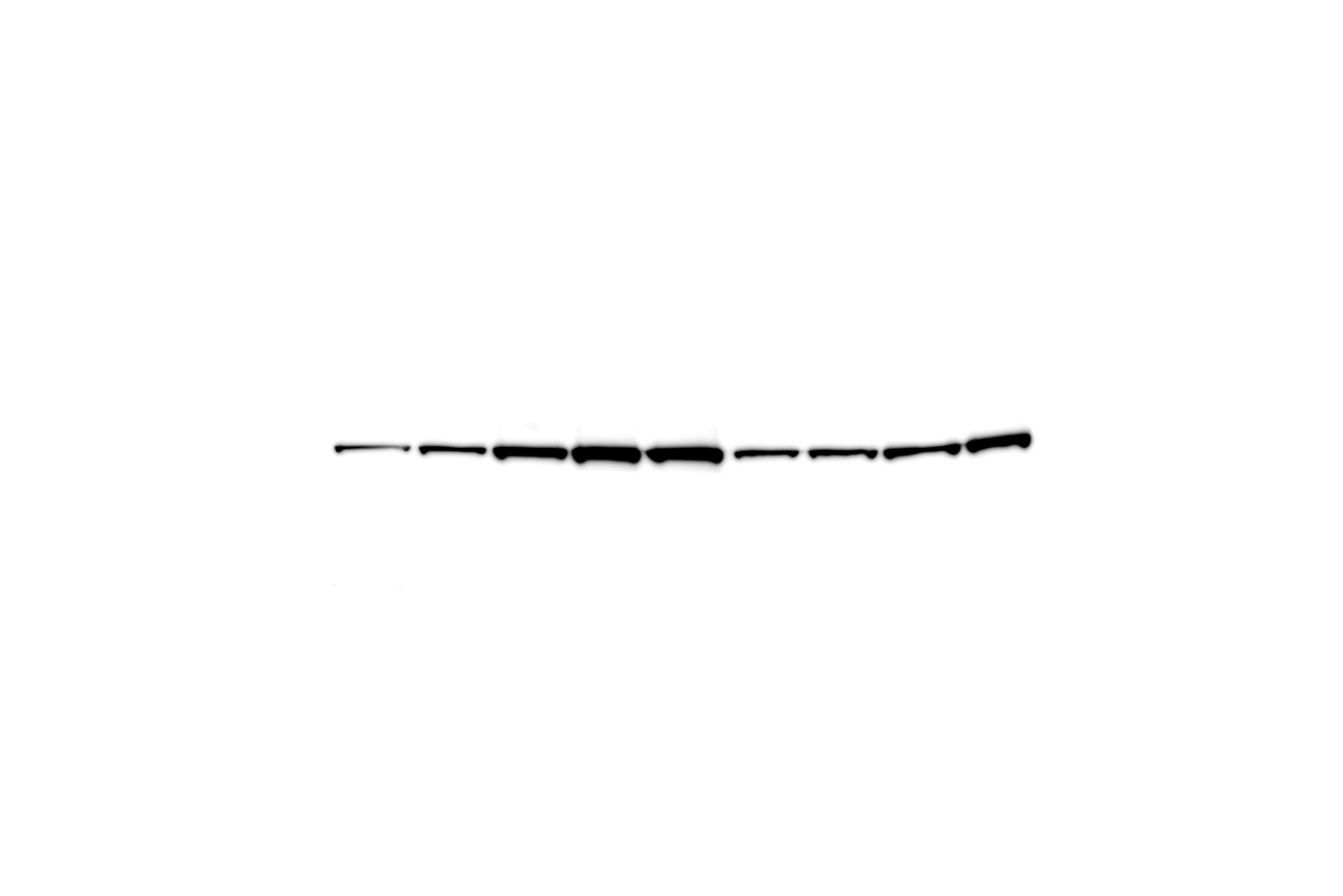


**FAS**





**FABP4**





**ACC**





**β-actin**

**Fig.3A**





**C\EBPβ**





**C\EBPα**





**PPARγ**





**FAS**





**β-actin**

**Fig.5H**





**Cyclin D1**





**β-actin**

**Fig.6H**





**PPARγ**





**C\EBPα**





**β-actin**





**Fig.7A**

**p-AMPK**





**AMPK**





**p-ACC**


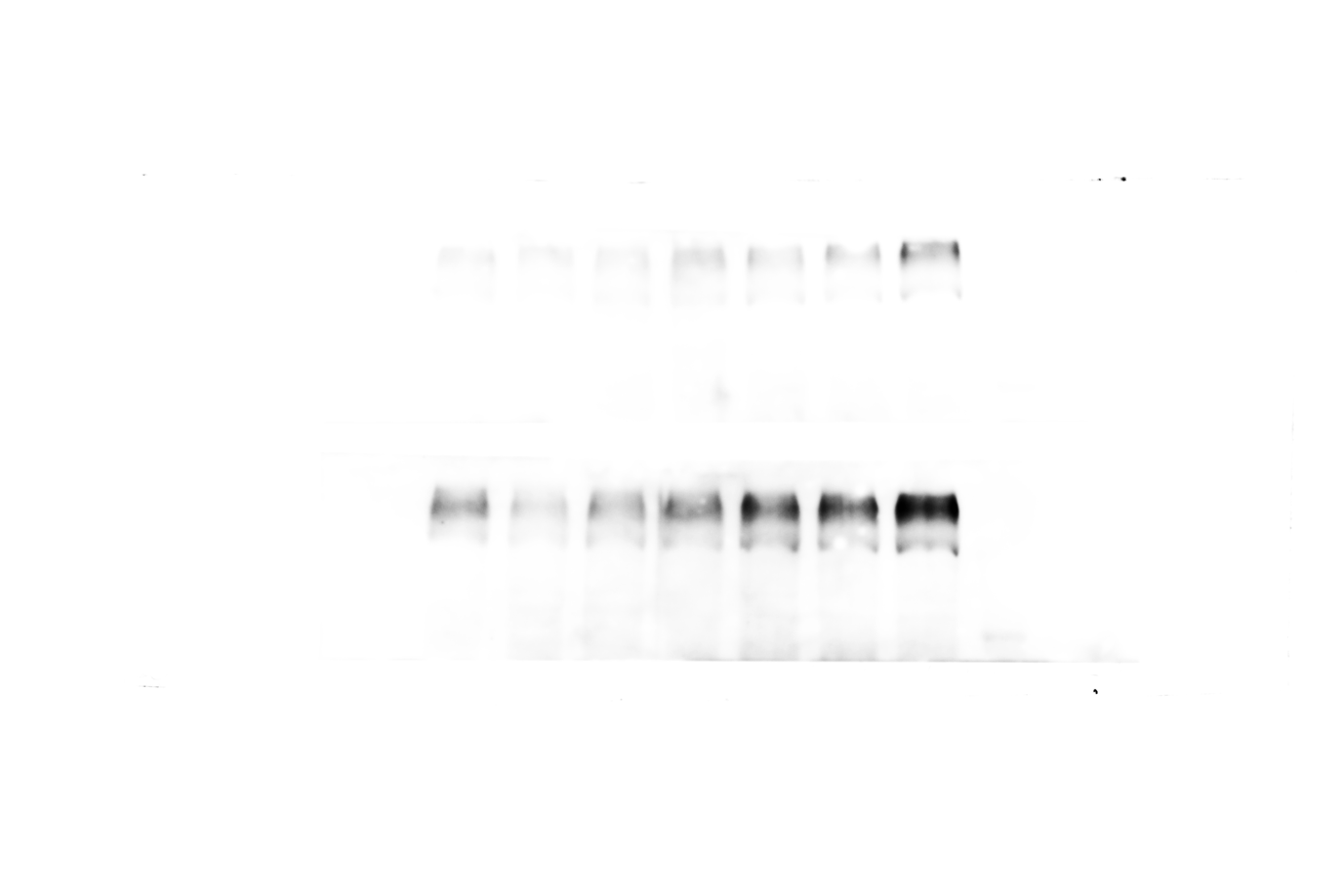


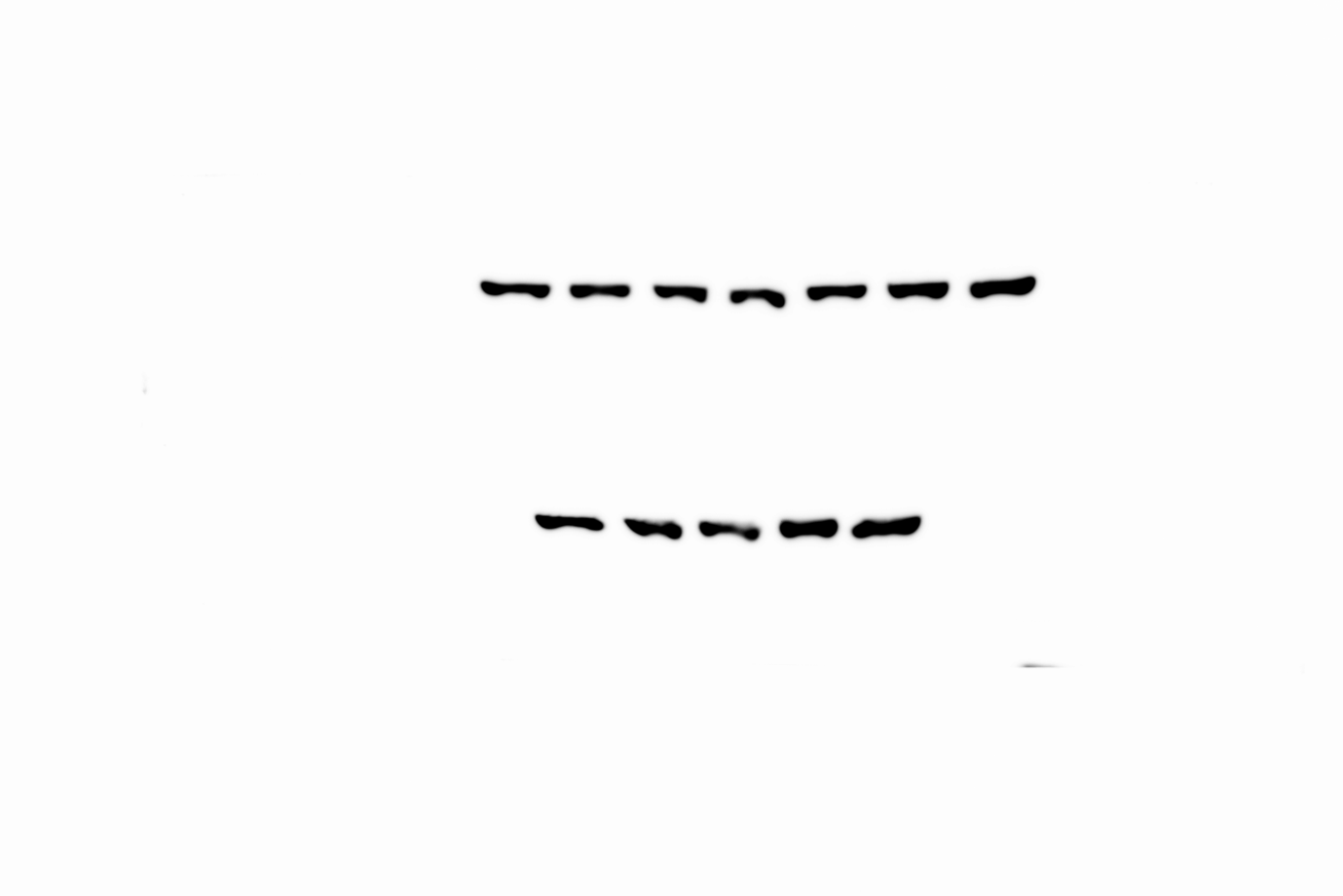


**ACC**





**β-actin**

**

Fig.7B**

**p-AMPK**

**
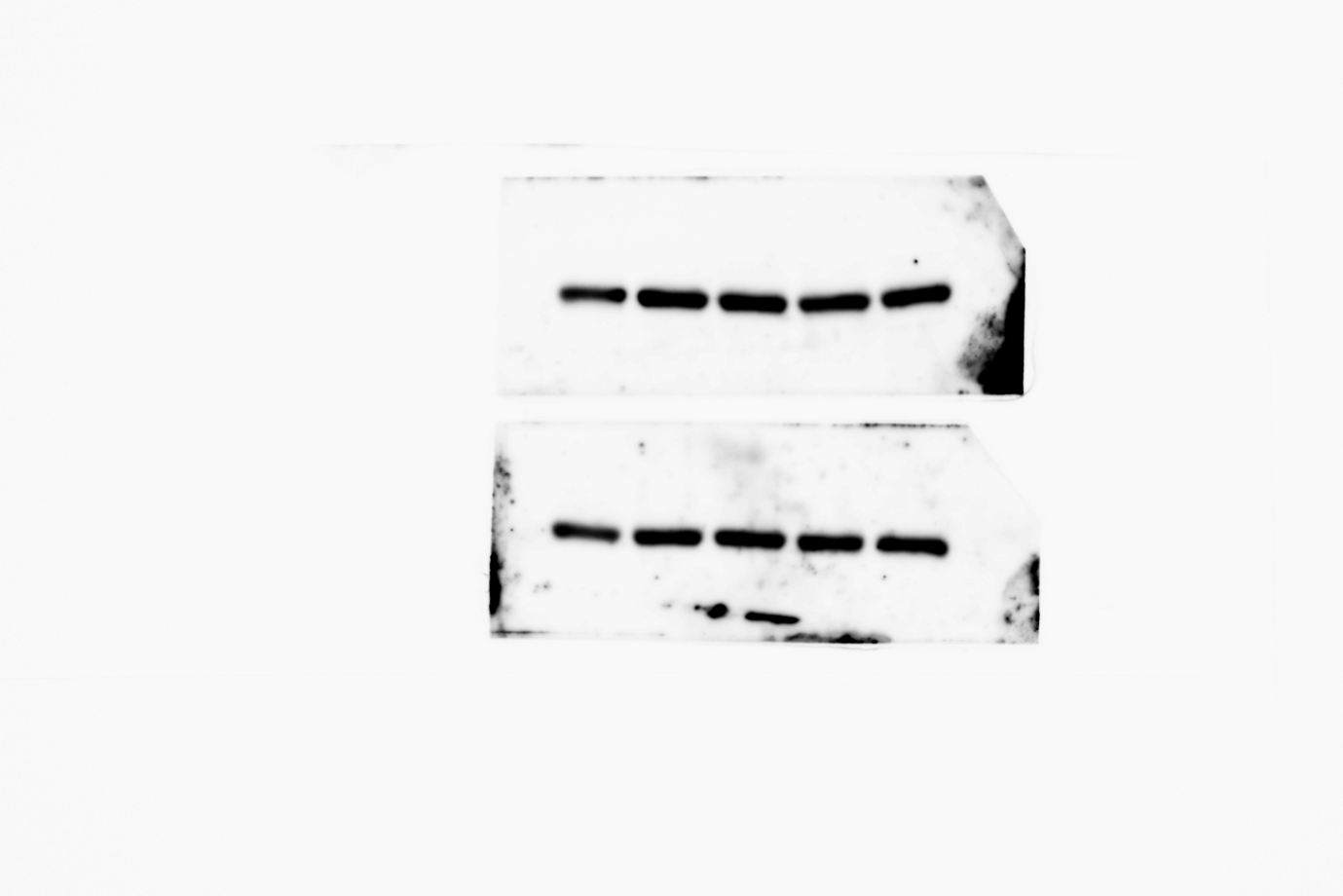
**

**AMPK**

**

**

**p-ACC**

**

**

**ACC**

**
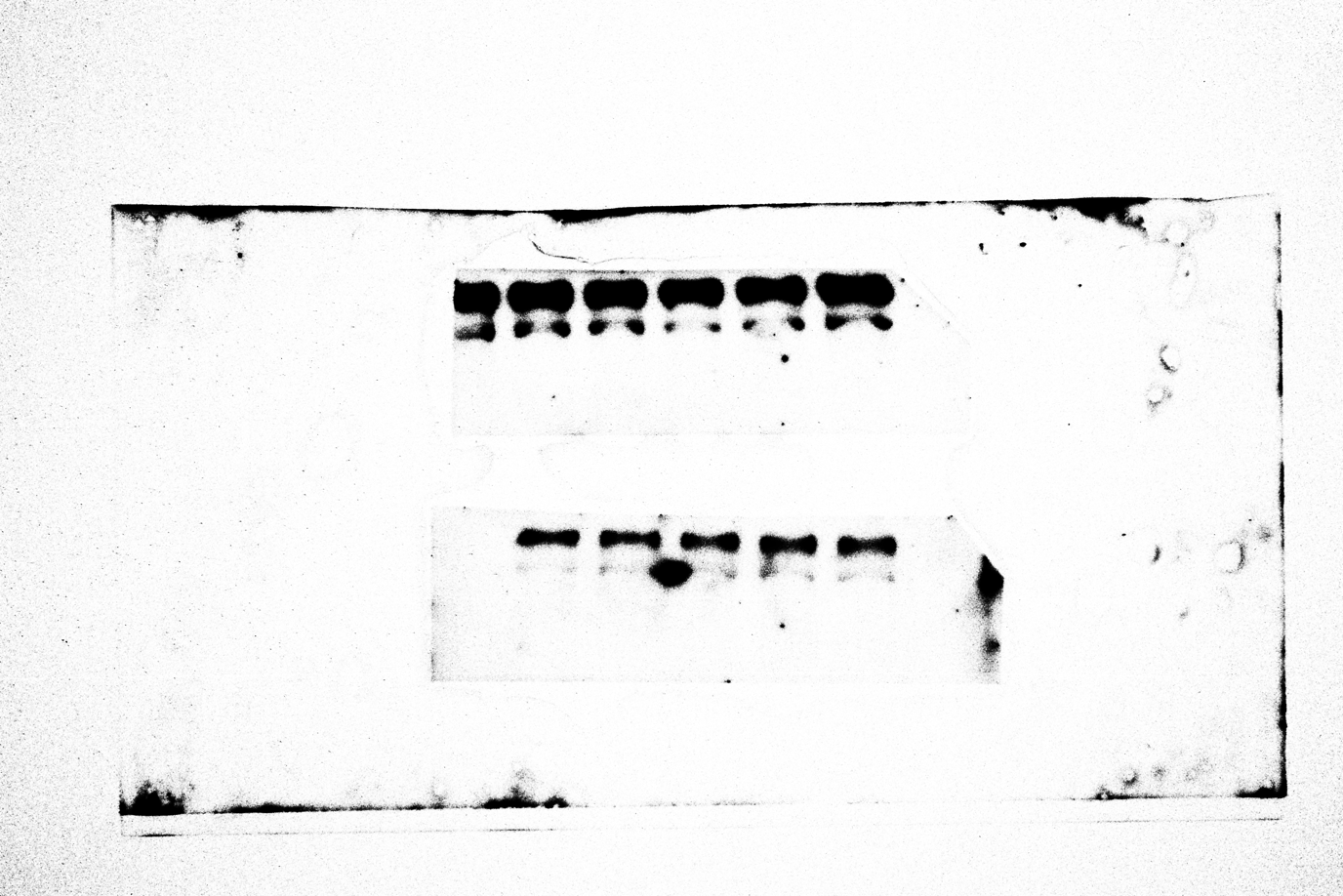
**

**

**

**β-actin**

**
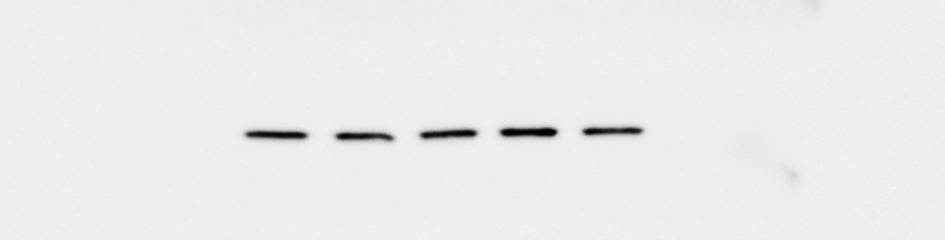
**

**Fig.S3**



**IκBα**





**β-actin**
